# Stepwise Development of an *in vitro* Continuous Fermentation Model for the Murine Caecal Microbiota

**DOI:** 10.3389/fmicb.2019.01166

**Published:** 2019-05-29

**Authors:** Sophie A. Poeker, Christophe Lacroix, Tomas de Wouters, Marianne R. Spalinger, Michael Scharl, Annelies Geirnaert

**Affiliations:** ^1^Laboratory of Food Biotechnology, Institute of Food, Nutrition and Health, ETH Zurich, Zurich, Switzerland; ^2^Department of Gastroenterology and Hepatology, University Hospital Zurich, Zurich, Switzerland

**Keywords:** microbiome, C57BL/6, mouse caecum, cultivation, *in vitro* model

## Abstract

Murine models are valuable tools to study the role of gut microbiota in health or disease. However, murine and human microbiota differ in species composition, so further investigation of the murine gut microbiota is important to gain a better mechanistic understanding. Continuous *in vitro* fermentation models are powerful tools to investigate microbe-microbe interactions while circumventing animal testing and host confounding factors, but are lacking for murine gut microbiota. We therefore developed a novel continuous fermentation model based on the PolyFermS platform adapted to the murine caecum and inoculated with immobilized caecal microbiota. We followed a stepwise model development approach by adjusting parameters [pH, retention time (RT), growth medium] to reach fermentation metabolite profiles and marker bacterial levels similar to the inoculum. The final model had a stable and inoculum-alike fermentation profile during continuous operation. A lower pH during startup and continuous operation stimulated bacterial fermentation (115 mM short-chain fatty acids at pH 7 to 159 mM at pH 6.5). Adjustments to nutritive medium, a decreased pH and increased RT helped control the *in vitro Enterobacteriaceae* levels, which often bloom in fermentation models, to 6.6 log gene copies/mL in final model. In parallel, the *Lactobacillus*, *Lachnospiraceae*, and *Ruminococcaceae* levels were better maintained *in vitro* with concentrations of 8.5 log gene copies/mL, 8.8 log gene copies/mL and 7.5 log gene copies/mL, respectively, in the final model. An independent repetition with final model parameters showed reproducible results in maintaining the inoculum fermentation metabolite profile and its marker bacterial levels. Microbiota community analysis of the final model showed a decreased bacterial diversity and compositional differences compared to caecal inoculum microbiota. Most of the caecal bacterial families were represented *in vitro*, but taxa of the *Muribaculaceae* family were not maintained. Functional metagenomics prediction showed conserved metabolic and functional KEGG pathways between *in vitro* and caecal inoculum microbiota. To conclude, we showed that a rational and stepwise approach allowed us to model *in vitro* the murine caecal microbiota and functions. Our model is a first step to develop murine microbiota model systems and offers the potential to study microbiota functionality and structure *ex vivo*.

## Introduction

The gastrointestinal tract of the mammalian host is inhabited by a dense, complex and diverse bacterial community, termed the gut microbiota (Robles Alonso and Guarner, 2013). The gut microbiota is involved in key processes beneficial for the host such as dietary compound metabolism, pathogen displacement, or immune system development (Round and Mazmanian, 2009; Robles Alonso and Guarner, 2013). A large proportion of the vast amount of evidence that the gut microbiota influences many physiological and pathological processes in the host comes from murine studies (Round and Palm, 2018). Mouse models are a valuable tool for human biology, disease and pharmaceutical research due to the high similarity in physiology, anatomy, and genetics (Nguyen et al., 2015). However, mice and humans differ in gastrointestinal tract anatomy, dietary habits, circadian rhythm, and environmental microbes and pathogens, which leads to differences in their gut microbiota composition and activity (Nguyen et al., 2015; Uhl and Warner, 2015; Hugenholtz and de Vos, 2017).

In humans, the large intestine is the main site of fermentation, and contains the highest microbial load and activity, whereas in mice, intestinal fermentation mainly takes place in the caecum, a “bag-like” link between the small intestine and the colon that is nearly absent in humans (Nguyen et al., 2015). Morphological differences in compartmentalization, retention times (RTs) and mixing conditions likely influences composition, richness and diversity of the gut microbiota in humans and mice (Nguyen et al., 2015). In both humans and mice, fermentation of indigestible food compounds produces the short-chain fatty acids (SCFAs) acetate, propionate and butyrate (Flint et al., 2012; Burokas et al., 2017; Herrmann et al., 2018). The bacterial composition influences the fermentation capacity and end microbial metabolite profiles (Flint et al., 2012). At phylum level, the murine and human intestinal microbiota are similar with the two dominating phyla Firmicutes and Bacteroidetes (Ley et al., 2006; Clavel et al., 2016; Hugenholtz and de Vos, 2017). However, when comparing the bacterial composition at a deeper phylogenetic level, mice and humans show clear differences in genera composition and abundance. First comparisons showed that 85% of detected bacterial genera in the mouse gut are not present in that of humans (Ley et al., 2005) and further studies identified mouse- and human-specific taxa (Nguyen et al., 2015; Xiao et al., 2015; Clavel et al., 2016). Genera such as *Lactobacillus, Turicibacter* and *Coprobacillus* (*Erysipelotrichaceae*), *Anaerotruncus* (*Ruminococcaceae*), *Marvinbryantia* (*Lachnospiraceae*), and *Pseudoflavonifractor* (unclassified *Clostridiales*) are present at higher levels in the murine intestinal microbiota, while *Prevotella, Faecalibacterium, Ruminococcus, Oscillibacter*, and *Klebsiella* are more abundant in human (Nguyen et al., 2015; Xiao et al., 2015). Deep metagenome sequencing revealed that only 4% of microbial genes in the mouse gut were shared with human gut samples, but almost 80% of their annotated functions were common (Xiao et al., 2015). This indicates a similar functionality of mouse and human gut microbiota, but performed by different species and strains.

Recently, efforts were made to isolate the murine gut bacteria (Lagkouvardos et al., 2016) to formulate minimal microbial consortia for studying microbiota-derived functions in gnotobiotic mouse models (Clavel et al., 2017). Simplified defined consortia may not recapitulate all functions of complex microbiota. Continuous *in vitro* fermentation models that contained the complex gut microbial communities mimicking the conditions of the modeled host were successful developed for ecological and mechanistic studies of human (Zihler et al., 2013; Dostal et al., 2015; Geirnaert et al., 2015; Lacroix et al., 2015; McDonald et al., 2015) and monogastric animal (Tanner et al., 2014) gut microbiota, which avoided ethical concerns and host confounding factors. However, to our knowledge no complex murine gut microbiota model has been developed, apart from simple, closed and short-term batch cultures (Salyer et al., 2013; Herrmann et al., 2017; Li et al., 2018) or continuous flow cultures without pH control and taxonomic validation (Freter et al., 1983). In contrast to batch cultures, continuous fermentation models offer the advantage of continuous substrate supply and removal of toxic fermentation products, which are required for growth and establishment of balanced and representative ecosystems. Physiological parameters like RT, temperature, pH, and redox potential are highly controlled in continuous fermentation, which facilitates the establishment of steady-state conditions (Payne et al., 2012). In addition, *in vitro* continuous gut fermentation models may allow simulation of the spatial, environmental and temporal features of a specific gut environment. Challenges of *in vitro* fermentation models include the large amount of gut microbiota sample (mainly feces) required for inoculation and the loss of less competitive bacteria by rapid washout. To address these challenges, fecal microbiota can be immobilized in porous gel beads and run in continuous culture, which allows models to operate at high cell density akin to the gut, prevents washout of slow growing bacteria, and reproduces both planktonic and sessile states of the gut microbiota (Zihler et al., 2013; Tanner et al., 2014; Fehlbaum et al., 2015; Poeker et al., 2018). In addition, only very small amounts of donor material (1–2 g) are required to establish a fermentation, therefore enabling modeling of small animal gut microbiota.

In this study, we developed a continuous fermentation model of the murine caecal microbiota based on the PolyFermS platform inoculated with immobilized caecal microbiota of C57BL/6 mice. In a first step, we determined the pH, the bacterial composition and the metabolic profile in the caeca of C57BL/6 mice. To support growth of the murine caecal bacterial populations and activity, a complex murine nutritive growth medium was developed to mimic substrate conditions encountered in mouse caecum chyme. Different factors of the fermentation model, including caecum sampling, fermentation starting mode, pH, RT and adjustment of growth medium were investigated and adjusted in a sequential order, using five different models inoculated with different pooled fresh immobilized caecum microbiota from 4-5 mice and operated up to 69 days. The microbiota composition in the reactor effluents was analyzed by quantitative real time PCR (qPCR) and 16S rRNA amplicon sequencing and compared with the caecal inoculum. Metabolic activity and functional stability of the microbiota was monitored by SCFA analysis.

## Materials and Methods

### Caecal Microbiota Collection

Healthy female WT C57BL/6J mice aged 9–13 weeks were obtained from Charles River (Lyon, France) and housed in groups at University Hospital of Zurich. Housing conditions were controlled at 22°C, room humidity and 12 h light/dark cycle. Mice were provided with mouse/rat maintenance chow (Kliba Nafag, Kaiseraugst, Switzerland) and *ad libitum* drinking water. Mice were housed in different cages and were sacrificed on the same day in the morning by cervical dislocation, after which the caecum was immediately removed and placed on a sterile petri plate. The pH of the fresh caecal content in the intact caecal pouch was immediately determined using a probe pre-calibrated pH meter (Metrohm 744 pH Meter, Metrohm Ltd., Herisau, Switzerland). The caecal content was collected into DNAase-free tubes, immediately snap frozen in liquid nitrogen, and then stored at -20°C until DNA and SCFA extraction. Mouse experiments were conducted according to Swiss animal welfare legislation, and the local veterinary office approved all procedures (Veterinäramt des Kantons Zürich; Nr. ZH220/2016).

### *In vitro* Fermentation Model

#### Nutritive Medium

The nutritive medium was formulated from the validated bacterial growth medium described by Macfarlane et al. (1998) for cultivating the human gut microbiota *in vitro*. It was modified to approximate the carbohydrate and protein ratio in murine caecum chyme. Standard mouse chow is composed of approximately 18% (w/w) protein, 54% (w/w) soluble carbohydrate and 4.5% (w/w) crude fiber (Supplementary Table S1B). Considering an average daily chow intake of 3.5 g per C57BL/6J mouse (Champy et al., 2008), a daily carbohydrate intake of 1.9 g, 0.6 g protein and 0.14 g crude fibers was considered. To calculate the amount of carbohydrates (excluding crude fibers) and proteins reaching the caecum, upper gastrointestinal digestibility indices of 95% for carbohydrates (Dahlqvist and Thomson, 1963a,b; Lee et al., 2011) and 90% for proteins (Kerr et al., 2014) were applied. This resulted in 0.09 g of dietary carbohydrates and 0.06 g of dietary proteins reaching the caecum each day or a dietary carbohydrate:protein ratio of 60:40. Macfarlane medium was adapted accordingly. (Supplementary Table S1A). To simulate the mouse chow more closely, we excluded guar gum and inulin, and replaced soluble potato starch with soluble corn starch. Concentrations of the protein sources casein, peptone, tryptone and yeast extract were increased to meet the calculated murine caecum chyme protein concentration. The mucin concentration was kept at 4 g/L. The pectin concentration was kept at 2 g/L to avoid flow disturbance in tubes from increased viscosity. The carbohydrate:protein ratio was approximately 60:40 in medium 1 and 55:45 in medium 2 (Supplementary Table S1A), which did not include the contribution of yeast extract and mucin used for simulating the endogenous protein and carbohydrate sources in the gastrointestinal tract (Cornick et al., 2015). After sterilization (20 min, 120°C) and cooling to 4°C, 1 mL of a filter-sterilized (0.2 μm pore-size) vitamin solution (Michel et al., 1998) was added to the medium. For initial bead colonization, the nutritive growth medium was supplemented with 20% (v/v) effluent from a previous fermentation (acetate:propionate:butyrate 2.5:1.5:1) or 20% (v/v) rumen fluid (4.5:3:1) and 0.1% (m/v) cellobiose (Table 1A). All components were purchased from Sigma-Aldrich Chemie (Buchs, Switzerland), except for xylane (Angene, London, United Kingdom), peptone water (Oxoid AG, Pratteln, Switzerland), bile salts (Oxbile, Oxoid AG), tryptone (BD, Sparks, United States), yeast extract (Merck, Darmstadt, Germany), NaHCO3 (Fischer Scientific, Pittsburgh, PA, United States), NaCl and KH2PO4 (VWR International AG, Dietikon, Switzerland), MgSO_4_⋅anhydrous (Acros Organics, Geel, Belgium) and MnCl_2_⋅4H_2_O (Fluka, Buchs, Switzerland).

**Table 1:** Conditions of initial batch (bead colonization) **(A)** and continuous **(B)** fermentation for the different tested models of mouse caecal fermentation.

(A) Batch conditions							
	**Model 1**	**Model 2**	**Model 3**	**Model 4**	**Model 5**	**Models 5^∗^**
Nutritive medium^∗^	6.8 g/L starch	6.8 g/L starch	6.8 g/L starch	2 g/L starch	2 g/L starch	2 g/L starch
Supplementations	20% effluent	10 mM lactate	0.1%cellobiose 20% rumen fluid	0.1%cellobiose 20% rumen fluid	0.1%cellobiose (Batch 1 and 2) 20% rumen fluid (Batch 1)	0.1%cellobiose (Batch 1 and 2) 20% rumen fluid (Batch 1)
PH	7	7	7	5.8	6	6
Fed-batch times						
Batch 1	24 h	24 h	16 h	20 h	20 h	20 h
Batch 2	24 h	24 h	5 h	6 h,	8 h	8 h
Batch 3	6 h	6 h				

**(B) Continuous fermentation conditions**							

	**Model 1**	**Model 2**	**Model 3**	**Model 4**	**Model 5**	**Models 5^∗^**

Nutritive medium^∗^	6.8 g/L starch	6.8 g/L starch	6.8 g/L starch	2 g/L starch	2 g/L starch	2 g/L starch
pH	7	7	7	6.5	6.5	6.5
Retention time	9 h	9 h	9 h	9 h	12 h	12 h
Total fermentation time (days)	13	44	32	53	69	42

*^∗^Composition presented in Supplementary Table S1.*

#### Caecal Microbiota Immobilization and Bead Colonization

For each immobilization procedure, four to five caeca from healthy 9- to 13- weeks old female C57BL/6 mice were collected to prepare the inoculum. All caecal contents were collected in the morning shortly (models 1 and 2) or a couple of hours (models 3–5) after the dark period. In model 1, caecal contents were dissected aerobically from the remaining gastrointestinal tract with surgical scissors, placed onto a sterile Petri dish and immediately transferred to an anaerobic box until further processing. In models 2–5, the caeca were tied off with surgical threads to avoid oxygen stress to the microbiota, placed onto a sterile Petri dish and immediately transferred to an anaerobic jar, and transferred into an anaerobic chamber (10% CO_2_, 5% H_2_, and 85% N_2_) (Coy Laboratories, Ann Arbor, MI, United States), for dissection and pooling contents. In models 3–5, caecal bacteria were washed with pre-reduced peptone water (0.1 %, pH 7, Thermo Fisher Diagnostics AG, Pratteln, Switzerland) before immobilization to remove potential interfering endogenous enzymes, salts and other cellular products that hinder proper gel bead formation. Caecal bacteria were immobilized in 1–2 mm gel beads consisting of gellan gum (2.5%, m/v), xanthan (0.25%, m/v), and sodium citrate (0.2%, m/v) in an anaerobic chamber as previously described (Poeker et al., 2018). Sixty milliliters of freshly produced caecal beads were transferred to a glass bioreactor containing 140 mL of sterile murine nutritive medium. For initial bead colonization, two or three consecutive fed-batch fermentations were carried out by replacing 100 mL of the spent medium by fresh nutritive medium every 6–24 h, depending on the model (Table 1A). The reactor headspace was continuously flushed with CO_2_, to ensure anaerobiosis in the system. The temperature was controlled at 37°C and the pH at selected values (depending on the model and fermentation step) by automatic addition of 2.5 M NaOH.

#### Continuous Caecum Microbiota Fermentation and Treatments

After batch fermentations, the reactor containing caecal beads was switched to continuous mode by continuously supplying fresh, sterile and anaerobic nutritive medium and removing an equivalent volume of fermented medium with peristaltic pumps (Reglo, Ismatec, Glattbrugg, Switzerland) (Table 1B). For all bioreactors, stirring speed was carried out at 180 rpm, working volume was 200 mL, temperature was set to 37°C and anaerobiosis was maintained by continuously flushing the headspace of bioreactors. In models 1–3, bioreactors were operated with constant conditions of pH 7 and a continuous flow rate of 22.2 mL/h (RT of 9 h) of medium 1 (6.8 g/L starch). Bioreactor in model 4 was maintained at pH 6.5 and supplied with fresh nutritive medium 2 (2 g/L starch) at a RT of 9 h. In model 5 and model 5^∗^, reactors were operated with constant conditions of pH 6.5 and a continuous flow rate of 16.7 mL/h (corresponds to a RT of 12 h) of medium 2. Effluent samples were taken daily and separated into bacterial pellets (10 min at 14,000 ×*g* at 4°C) and supernatant, and stored at -20°C until further analysis. Stability of *in vitro* microbiota was monitored by daily measurements of the main fermentation metabolite concentrations in sample supernatants.

### Microbial Metabolite Analysis

Caecal samples were mixed with 300 μL 0.15 mM H_2_SO_4_, homogenized and centrifuged at 4°C at 9000 ×*g* for 20 min. Supernatant was filtered directly into HPLC vials through a 0.45 μm nylon membrane (Infochroma AG, Zug, Switzerland). Fermentation samples were centrifuged at 4°C at 14,000 ×*g* for 10 min. The pellet was used for DNA extraction and the supernatant was filtered into glass vials through a 0.45 μm nylon membrane. HPLC analysis (Thermo Fisher Scientific Inc. Accela, Wohlen, Switzerland) was performed to determine the concentrations of SCFAs (formate, propionate, acetate, butyrate, and valerate), branched-chain fatty acids (BCFAs) (isobutyrate and isovalerate) and intermediate metabolites (lactate and succinate). Analyses were performed with an Accela Chromatography System and RI-detector (Thermo Fisher Scientific Inc., Reinach, Switzerland), equipped with a Security Guard Carbo-H cartridge (4 × 3.0 mm) and a Rezex ROA-Organic Acid H^+^ column (300 × 7.8 mm). Volumes of 40 μL were injected into the HPLC with a flow rate of 0.4 mL/min and H_2_SO_4_ as an eluent (Poeker et al., 2018).

### Microbial Community Analysis

#### Genomic DNA Extraction

Total genomic DNA was extracted from 100 to 200 mg of caecal contents and the pellet of 2 mL of fermentation effluent using the FastDNA^®^ SPIN Kit for Soil (MP Biomedicals, Illkirch Cedex, France) according to the manufacturer’s instructions. Total DNA concentration was measured by spectrophotometry using a Nanodrop^®^ND-1000 Spectrophotometer (Wiltec AG, Littau, Switzerland) and samples were stored at -20°C until analysis.

#### Quantitative PCR Analysis

DNA extracts were used for qPCR to enumerate the gene copy numbers of total bacteria and the specific marker bacterial groups Firmicutes, *Ruminococcaceae*, *Lachnospiraceae*, *Lactobacillus*–*Leuconostoc*–*Pediococcus*, Bacteroidetes, *Bacteroides*, *Enterobacteriaceae*, and *Akkermansia* (Supplementary Table S2). qPCR assays were performed using 1 μL of 10- or 100- fold diluted genomic DNA, 2x SYBR Green qPCR Mastermix (Life Technologies, Labgene Scientific Instruments, Zug, Switzerland), 100 μM of each forward and reverse primer, resulting in a total volume of 25 μL in a 96-well plate. The analysis was performed in an ABI PRISM 7500-PCR -sequence detection system (Applied Biosystems, Zug, Switzerland). Each reaction was run in duplicate. For quantification, a dilution series of standards was obtained by amplification of the linearized plasmids containing the gene of a representative bacterial species belonging to the target group, and included in each run (Pham et al., 2017). Primer specificity and verification of the presence of the desired amplicon was determined by melting curve analysis. PCR efficiency (%) was calculated from the slope of the standard curve of each qPCR assay. Assays with an efficiency of 80–110% (slope of 3.2–3.9) were included in data analysis.

#### Microbiota Profiling With 16S rRNA Amplicon Sequencing

The bacterial community in caecal samples was analyzed using the primers 341F (5′-CCTAYGGGRBGCASCAG-3′) and 806R (5′-GGACTACNNGGGTATCTAAT-3′), which flank the V3-V4 region. MiSeq adaptors were added by PCR. Sequencing of caecum microbiota of different mice was performed with Illumina MiSeq (Genotoul, Castanet-Tolosan Cedex Mainz, France). For the microbiota analysis of caecal inocula and fermentation samples of the final models 5 and 5^∗^, the V4 region of the 16S rRNA gene was amplified with primers 515F (5′-GTGCCAGCMGCCGCGGTAA-3′) and 806R (5′-GGACTACHVGGGTWTCTAAT-3′). Sequencing was performed with Illumina MiSeq (Genomic Diversity Centre, ETH Zurich, Zurich, Switzerland) with V2 reagent kit for 2 × 250 bp paired end Next Tera chemistry supplemented with 20% of PhiX. The raw sequence data has been submitted to European Nucleotide Archive (ENA) database with accession number PRJEB30419. The open-source bioinformatics pipeline Quantitative Insight Into Microbial Ecology (QIIME) (Caporaso et al., 2010) was used to process the raw 16S rRNA gene sequencing data. The raw dataset containing pair-ended reads with corresponding quality scores were merged and trimmed using fastq_mergepairs and fastq_filter scripts implemented in the UPARSE pipeline (Edgar, 2013) as previously described (Krych et al., 2018). The minimum overlap length of trimmed reads was set to 50 bp (V4) or 20 bp (V3V4). The minimum length of merged reads was 180 bp (V4) or 200 bp (V3V4), the max expected error *E* = 2.0, and first truncating position with quality score *N* ≤ 4. Purging the dataset from chimeric reads and constructing *de novo* Operational Taxonomic Units (OTU) were conducted using the UPARSE pipeline (Edgar, 2013). The green genes 16S rRNA gene collection (version 13.8) was used as a reference database (Werner et al., 2012) and an OTU count table including taxonomy was generated. The OTUs assigned to the S24-7 family were reported in the manuscript as taxa of the *Muribaculaceae* family (Lagkouvardos et al., 2016). QIIME open source software package (1.8.0 and 1.9.0) was used for subsequent analysis, including alpha and beta diversity, and the PICRUSt (phylogenetic investigation of communities by reconstruction of unobserved states) (Langille et al., 2013) analysis to predict the KEGG metabolic pathways and COG functional groups from microbiota samples.

#### Statistical Analysis

To compare SCFA concentrations between fermentation models, statistical tests were carried out using SigmaPlot 13.0 (SigmaPlot Software, La Jolla, CA, United States). Significance level was set at 0.05. Normality of the data set was tested with the Kolmogorov–Smirnov test. In case of normality, mean values of two different groups were compared with an independent samples *t*-test. In case of non-normality, differences were tested with non-parametric Mann–Whitney *U* test. Data are expressed as means ± standard deviations (SD) of 3 days of the stabilization and treatment periods with standard observed variations of metabolites lower than 10% in the same reactor in each fermentation. To assess if there were significant differences in taxa abundance between caecal inoculum and PolyFermS microbiota, or between different PolyFermS microbiota, the DESeq2 method (Love et al., 2014) was used on the unnormalized count data as previously suggested by McMurdie and Holmes (2014).

## Results

In the first step, caecal physiological parameters such as pH, bacterial fermentation metabolites and composition were investigated for the C57BL/6 mouse strain. This breed was selected to inoculate the fermenters because it is the most widely used strain in biomedical and gut microbiota research (Bryant, 2011). To establish the *in vitro* murine caecal microbiota model, we analyzed fermentation metabolites by HPLC and quantified specific bacterial populations, particularly the stress- and oxygen-sensitive families *Lachnospiraceae* and *Ruminococcaceae*, and the genus *Lactobacillus* by qPCR as main markers for representative bacterial groups of murine gut microbiota. Overgrowth of the *Enterobacteriaceae* family was also monitored to detect a potential lack of control of oxygen stress during sampling and start-up of fermentation. These microbial indicators were used to suggest subsequent changes during the sampling, bead colonization and continuous fermentation process to achieve a balance akin to the mouse caecum content used for immobilization and inoculation of the continuous fermentation model. The final conditions set for model 5 were repeated with two caecal inocula prepared from different mice (model 5^∗^).

### Composition of Caecal WT C57BL/6 Mouse Microbiota and Metabolites

**FIGURE 1:**
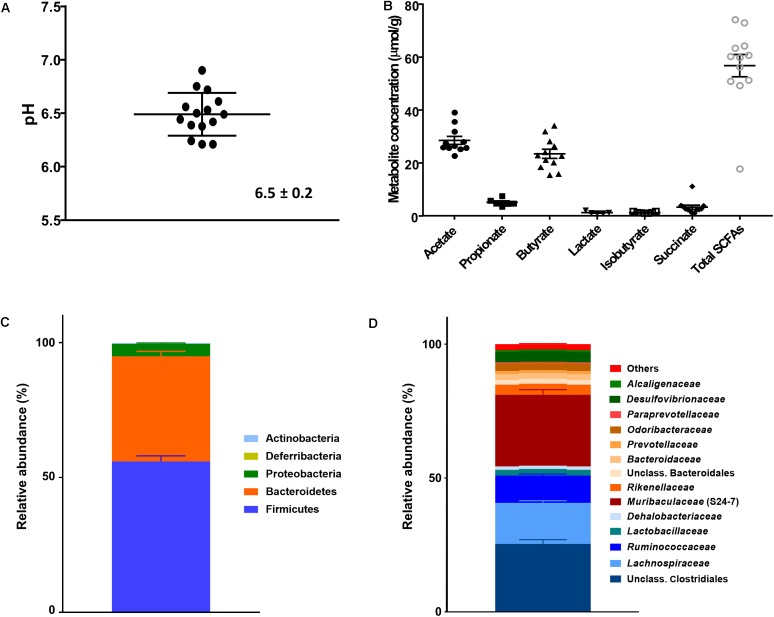
Analysis of caecal contents of WT C57BL/6 mice (means ± SEM; *n* = 15). **(A)** pH; **(B)** Metabolite concentrations (μmol/g); **(C)** Microbial composition obtained by 16S rRNA amplicon sequencing and expressed as relative abundance at phylum and **(D)** family level. Data are mean ± SEM. Values < 1% are summarized in the group «Others».

Caecal pH in WT C57BL/6 mice (*n* = 15) ranged from 6.2 to 6.9 with a mean of 6.5 ± 0.2 (Figure 1A). Caecal fermentation metabolites; the SCFAs acetate, propionate and butyrate; the intermediates succinate and lactate; and the BCFA isobutyrate, were detected in all murine caeca (Figure 1B). Important individual variations of total metabolite concentration (17.7–74.1 μmol/g; average of 56.7 ± 14.6 μmol/g), and of the main end metabolites acetate (28.5 ± 5.0 μmol/g), propionate (5.1 ± 1.4 μmol/g) and butyrate (23.5 ± 5.9 μmol/g) were measured, while succinate (between 1 and 11 μmol/g), lactate (1–2 μmol/g), and isobutyrate (1–2 μmol/g) were detected at lower concentrations. The average acetate:propionate:butyrate ratio was 50:9:41, which was in the range of previous reports in same mouse strain on similar chow (Krautkramer et al., 2017). Caecal microbiota were dominated by bacterial phyla Firmicutes (56.0 ± 8.0%) and Bacteroidetes (38.9 ± 7.3%), and also harbored Proteobacteria at lower levels (4.6 ± 1.3%) (Figure 1C). Within Bacteroidetes, *Muribaculaceae* (S24-7) and *Rikenellaceae* were the most abundant families, and within the Firmicutes an unclassified Clostridiales family, *Lachnospiraceae* and *Ruminococcaceae* (Figure 1D). The α-diversity Shannon index (H) ranged from 5.6 to 6.2 (average 5.9 ± 0.9) (data not shown). Principal Coordinate Analysis (PCoA) was used to analyze β-diversity to characterize the degree of individual variations among the caecal murine microbial communities. Mice of the same age co-housed within the same cage for 21 days or in different cages were analyzed. The caecal microbiota of mice housed in the same cage did not cluster and individual mouse microbiota scattered in both unweighted and weighted Unifrac distance PCoA (Supplementary Figure S1), indicating a large inter-individual variation in caecal microbiota composition.

### Development of *in vitro* Model for Murine Caecal Microbiota Fermentation

During bead colonization, the effects of pH in combination with different nutritive media as well as initial batch fermentations conditions for bead colonization were assessed. During continuous operation, the impact of pH in combination with different RTs were investigated to improve maintenance of activity and composition of the murine caecal microbiota *in vitro* and compare to *in vivo* caecum data.

**FIGURE 2:**
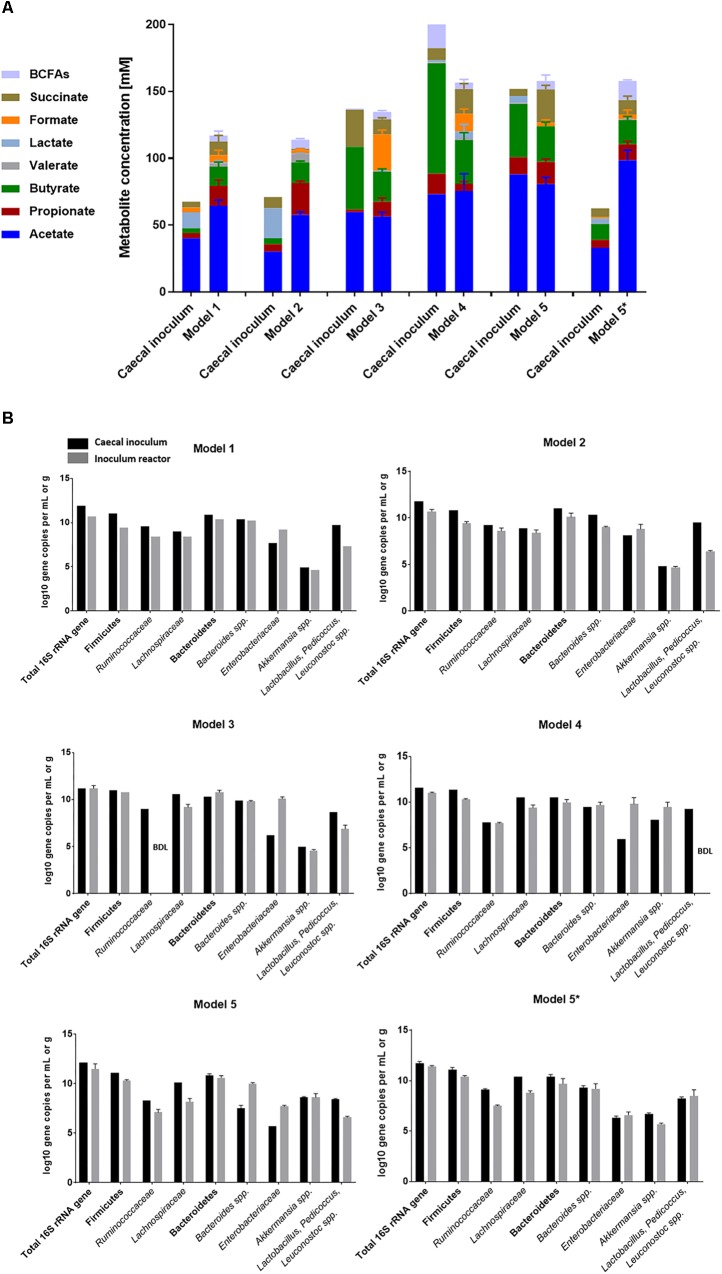
Bacterial activity and composition of caecal inocula and reactor effluents of different models. **(A)** Concentrations of metabolites (mM) in caecal inocula and reactor effluents of stabilization phases expressed as mean metabolite concentrations with standard error. **(B)** Quantification of key bacterial populations in caecal inocula and fermentation samples of different models by qPCR and expressed as means ± SD log gene copies/g or mL when *n* > 1. BDL, below detection limit of 4 log10 gene copies.

In model 1, caecal content sampling and processing was performed rapidly but under aerobic conditions and bead colonization was done at pH 7 in nutritive medium 1 supplemented with 20% (v/v) fermentation effluent during three batch cultures (Table 1A). During continuous operation, constant conditions of pH 7 and RT 9 h were used. Metabolic stability was observed after 4 days of continuous fermentation with a mean total metabolite concentration of 115 ± 10 mM from day 4 to day 12 (Figure 2A and Supplementary Figure S2A). Acetate (65 ± 4 mM) was the main produced metabolite, followed by butyrate (14 ± 3 mM) and propionate (15 ± 4 mM) (Supplementary Table S3). We observed higher levels of lactate, an intermediate fermentation metabolite, in the caecal inoculum compared to the first sampled caecal contents (Figure 1B) and compared to *in vitro* levels (Figure 2A). This might be because caecal contents for model 1 were sampled in the morning shortly after dark period and it was previously observed that lactate levels were then highest (Hamaguchi et al., 2015; Tahara et al., 2018). At the bacterial compositional level, a decrease in butyrate-producing families *Ruminococcaceae* (-1.2 log gene copies) and *Lachnospiraceae* (-0.6 log gene copies) was observed compared to the corresponding caecal inoculum (Figure 2B and Supplementary Table S4). *Lactobacillus* spp. were also decreased *in vitro* compared to the caecal inoculum (-2.4 log gene copies vs. *in vivo*), while *Enterobacteriaceae* were increased (+1.5 log gene copies vs. *in vivo*).

We assumed that the short exposure to oxygen during sample collection favored the growth of facultative anaerobes, including *Enterobacteriaceae* and was detrimental to strict anaerobes such as bacterial taxa within Clostridiales. Therefore, in model 2 the sampling procedure was adapted to better protect the microbiota from oxygen. In addition, the batch fermentation medium was supplemented with lactate to enhance the growth and activity of lactate-consuming butyrate-producers within the bacterial order Clostridiales (Bourriaud et al., 2005; Tao et al., 2016). Metabolic stability of model 2 was reached after 12 days of continuous operation (Figure 2A and Supplementary Figure S2B) with mean total metabolite production of 114 ± 3 mM. In model 2, an acetate:propionate:butyrate (AA:PA:BA) ratio of 61:24:15 was observed, with slightly increased propionate levels compared to the caecal inoculum (73:15:12) (Supplementary Table S3). Compared to the caecal microbiota inoculum, *Enterobacteriaceae* (+0.7 log gene copies) were increased in model 2 effluents, while *Lactobacillus* spp. (-3.1 log gene copies) and butyrate-producers (*Lachnospiraceae*: -0.5 log gene copies; *Ruminococcacea*e: -0.6 log gene copies) were decreased (Figure 2B and Supplementary Table S4).

The medium of model 3 was supplemented with both cellobiose and rumen fluid to improve bead colonization of butyrate-producing bacteria and to enhance the growth of *Lactobacillus* spp. Rumen fluid contains primary nutrients for cross-feeding and microbial growth factors (Bryant, 1959; Kamada et al., 2013; van Zanten et al., 2014). In addition, the number of bead colonization batch fermentations was reduced to two, and the duration for the first batch was extended to 16 h to decrease the growth advantage of fast-growing *Enterobacteriaceae*, promote growth and activity of butyrate-producing bacteria (Clostridia), and achieve a more complete carbohydrate fermentation with high re-utilization of intermediate metabolites (lactate, formate, acetate, succinate). Metabolic stability of model 3 was reached after 15 days, with a higher mean total metabolite concentration (135 ± 4 mM) compared to model 2 (*p* < 0.05) and model 1 (*p* < 0.05) and a AA:PA:BA molar ratio of 63:12:25 (Figure 2A, Supplementary Table S3, and Supplementary Figure S3A). However, concentrations of the butyrate-producing families *Lachnospiraceae* (-1.1 log gene copies vs. *in vivo*) and *Ruminococcaceae* (below detection limit) remained low compared to caecal inoculum. Further, lower levels of *Lactobacillu*s spp. (-1.8 log gene copies) and higher levels of *Enterobacteriaceae* (+3.9 log gene copies) were detected in the effluent samples compared to the caecal inoculum (Figure 2B and Supplementary Table S4).

Other possible strategies to prevent the outgrowth of *Enterobacteriaceae* are reducing the concentration of simple carbohydrates in the nutritive medium (here: corn starch), and decreasing the pH (from pH 7 to 5.8 during batch fermentation and from pH 7 to 6.5 during continuous operation), since optimal growth pH of these bacteria is close to neutrality (Supplementary Table S1 and Table 1). After 19 days of continuous culture, steady metabolite production was reached with higher total metabolite production (154 ± 13 mM) compared to previous model 3 (*p* < 0.05) and a AA:PA:BA ratio of 66:4:29 (Figure 2A, Supplementary Table S3, and Supplementary Figure S3B). High butyrate levels were observed in caecal inoculum (83 mM) and reactor effluent (33 mM), which can be associated with high and comparable levels of butyrate-producing families *Ruminococcaceae* (7.7 ± 0.1 log gene copies/mL and 7.5 log gene copies/g, respectively) and *Lachnospiraceae* (9.4 ± 0.3 log gene copies/mL and 10.5 log gene copies/g, respectively; Figure 2B and Supplementary Table S4). However, the concentration of *Enterobacteriaceae* remained high (+3.8 log gene copies/mL compared to caecal inoculum) and *Lactobacillus* spp. remained below the detection limit.

A recent study found that some bacterial populations within Clostridiales order are positively associated with long RTs in humans, which may also promote growth conditions for the slow-growing bacterial populations in our model (Roager et al., 2016). Therefore, in model 5 we assessed whether an increase in RT from 9 h (model 4) to 12 h can prevent overgrowth of fast-growing *Enterobacteriaceae*. A high total metabolite production was obtained (158 ± 9 mM) with higher levels of propionate (*p* < 0.05) and lower levels of the intermediate metabolite formate (*p* < 0.05) compared to model 4 (Figure 2A, Supplementary Figure S4, and Supplementary Table S3). The AA:PA:BA ratio *in vitro* (65:14:22) was comparable with the ratio detected in the caecal inoculum (62:9:28). Concentrations of potential butyrate-producing bacterial markers *Lachnospiraceae* (8.2 ± 0.3 log gene copies/mL vs. 10.1 ± 0.0 log gene copies/g) and *Ruminococcaceae* (7.1 ± 0.3 log gene copies/mL vs. 8.3 ± 0.0 log gene 372 copies/g) were lower in effluent samples compared with caecal inoculum levels (Figure 2B and Supplementary Table S4). Despite this, increased retention resulted in less overgrowth of *Enterobacteriaceae* (5.7 ± 0.1 log gene copies/g caecal content vs. 7.7 ± 0.1 log gene copies/mL model 5 effluent) and no severe loss of *Lactobacillus* spp. (8.4 ± 0.1 log gene copies/g caecal content vs. 6.6 ± 0.1 log gene copies/mL model 5 effluent) compared to model 4.

As model 5 conditions reflected the metabolic and bacterial concentrations of the mouse caecum adequately, an independent repetition of model 5 was performed (starting from another caecal microbiota inoculum; referred to as model 5^∗^). The total fermentation metabolite production (161 ± 7 mM) was comparable to levels in model 5 (Figure 2A and Supplementary Table S3). Overall, the metabolite production in both model 5 and 5^∗^ was stable during continuous operation of 69 and 43 days, respectively (Supplementary Figures S4, S5). At the bacterial marker level, comparable concentrations of *Bacteroides* spp. and *Akkermansia* spp. were detected in model 5^∗^ effluent samples compared to its caecal inoculum (Supplementary Table S4). Interestingly, the *in vitro Akkermansia* spp. levels (5.7 ± 0.1 log gene copies/mL) reflected the lower concentrations present in caecal inoculum (6.7 ± 0.1 log gene copies/g in caecal inoculum 5^∗^ vs. 8.6 ± 0.1 log gene copies/g in caecal inoculum 5). *Enterobacteriaceae* spp. (6.6 ± 0.3 log gene copies/mL) and *Lactobacillus* spp. (8.5 ± 0.6 log gene copies/mL) established at comparable levels to those detected in the caecal inoculum (6.3 ± 0.1 log gene copies/g and 8.2 ± 0.2 log gene copies/g, respectively).

### Microbiota Analysis of Final *in vitro* Continuous Fermentation Murine Caecum Model

**FIGURE 3:**
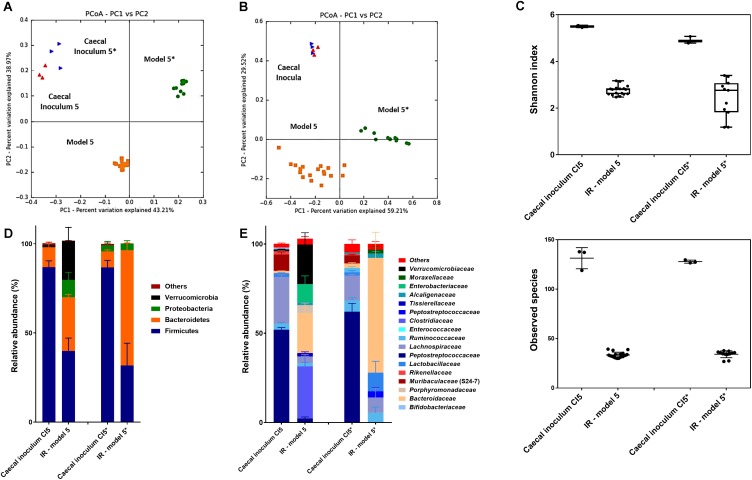
Microbial composition and diversity analysis in caecal inocula and reactor effluents of model 5 and 5^∗^. **(A,B)** Principle Coordinate Analysis (PCoA) of caecal inocula and reactor microbiota based on unweighted **(A)** and weighted **(B)** UniFrac analysis matrix on OTU level. Each point represents a microbiota sample from murine caecal content used as inoculum for model 5 (red 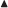) and model 5^∗^ (blue 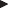) or from stabilized reactor effluents of model 5 (orange 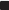) and model 5^∗^ (green 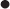). **(C)** Alpha diversity measured by Shannon diversity index and observed species. The box depicts distribution of diversity index for caecal inocula and fermentation samples. Boxes represent the interquartile range (IQR) between the 5th and 95th percentiles, respectively. **(D,E)** Microbial composition in caecal samples of WT C57BL/6 mice obtained by 16S rRNA amplicon sequencing. Relative abundance at phylum and family level of caecal inocula and fermentation samples of model 5 and 5^∗^. Data are mean ± SD. Values < 1% are summarized in the group «Others».

To assess the overall microbiota composition and diversity in comparison to the caecal inocula, reactor effluent microbiota of model 5 and 5^∗^ were further analyzed by 16S rRNA gene sequencing. The composition of reactor microbiota differed from the caecal inoculum microbiota as indicated in a PCoA-biplot on weighted and unweighted UniFrac distance (Figure 3A,B). Furthermore, model 5 microbiota differed from model 5^∗^ microbiota as shown by a spatial separation and clustering in both unweighted and weighted UniFrac PCoA biplots. The two caecal inocula microbiota showed scattering in the unweighted UniFrac PCoA, indicating qualitative differences in their composition.

The bacterial diversity within the reactor microbiota was lower compared to the caecal inocula microbiota (Figure 3C). Both the Shannon diversity-index that takes into account the number of observed OTUs and their relative evenness and the observed species diversity decreased from 5.2 for caecal inocula to 2.7 ± 0.6 for reactor effluent samples and from 130 to 35, respectively.

Overall abundance shifts from *in vivo* to *in vitro* caecal microbial communities occurred (Figure 3D,E). Compared to the caecal inocula, the Bacteroidetes phylum abundance increased while Firmicutes phylum abundance decreased in both *in vitro* microbiota; this shift was most pronounced in model 5^∗^ (Figure 3D and Supplementary Table S5). Certain genera and species from the caecal inoculum flourished *in vitro*, while others became established at a lower abundance or below the detection limit. This resulted in significant and large log2 fold changes in abundance when comparing caecal inoculum to corresponding fermentation samples using DeSeq2 analysis (Supplementary Figures S6, S7). Taxa from *Bacteroides, Anaerococcus* and unclassified *Clostridiaceae* genera were enriched *in vitro* in both models with a log2 fold change exceeding 4. Some abundant (>1%) taxa in the caecal inocula established at a lower abundance (unclassified Clostridiales and *Lactobacillus* genera) or were not detected (taxa from *Muribaculaceae* (S24-7) family).

Compositional differences between the two *in vitro* microbiota were observed, which were in line with the quantitative differences detected by qPCR (Supplementary Figure S8). Model 5 *in vitro* microbiota was characterized by taxa belonging to *Akkermansia*, *Enterobacteriaceae*, *Parabacteroides*, and *Clostridiaceae*, while in model 5^∗^, *in vitro* microbiota taxa belonging to *Lactobacillus*, *Peptostreptococcaceae, Blautia*, and *Anaerofilum* established better compared to model 5.

We next predicted the gene content from the 16S rRNA sequence data by PICRUSt (phylogenetic investigation of communities by reconstruction of unobserved states) for revealing potential functional differences between both *in vitro*- and caecal inocula microbiota. Conserved metabolic and functional KEGG pathways were observed in both microbiota types (Supplementary Figure S9), indicating a similar microbial functional potential between *in vitro*- and caecal inocula microbiota.

## Discussion

In this study, we aimed to develop a continuous *in vitro* fermentation model that reflects the metabolic activity and phylogeny of healthy WT mouse caecal microbiota. An important prerequisite for *in vitro* studies is the rational selection of models and conditions, while keeping in mind that these models can never completely represent reality (Lacroix et al., 2015). Therefore, we followed a stepwise approach for model development by adjusting parameters to reach fermentation metabolite profiles and marker bacterial levels similar to the *in vivo* situation.

During continuous operation a stable *in vitro* fermentation with main fermentation metabolites acetate, propionate and butyrate was reached, which is in line with *in vivo* measurements in this and other studies reporting ratios of murine caecal microbial fermentation metabolites (Burokas et al., 2017; Krautkramer et al., 2017; Herrmann et al., 2018). *In vivo*, the SCFA are continuous and efficiently absorbed by the intestinal epithelium (Morrison and Preston, 2016), resulting in an underestimation of the actual caecal fermentation capacity based on caecal SCFA measurements. Accordingly, the total SCFA concentrations *in vitro* were higher than *in vivo* because absorption is not simulated. Hence, model values reflect the total fermentation capacity of the modeled caecal microbiota. First models showed a limited fermentation capacity, but thanks to the optimization steps the total metabolite production increased from 115 mM in model 1 to 159 mM and 161 mM in model 5 and 5^∗^, respectively. Along with the improved fermentation capacity, a simultaneous longer stabilization time of the models was observed; this might be explained by the growth and balance of a more complex microbiota reliant on cross-feeding mechanisms. A lower pH during startup and continuous operation (models 3–4) stimulated bacterial fermentation as previously observed in human microbiota fermentation models (Walker et al., 2005; Zihler et al., 2013). Increased RT (model 4–5) resulted in decreased accumulation of the intermediate metabolites formate and lactate, and higher levels of branched SCFA, which are specific markers for protein fermentation and associated with long RTs (Davila et al., 2013; Tottey et al., 2017). Succinate was after acetate, propionate and butyrate the microbial metabolite detected at the highest concentrations in all caecal murine fermentation models and their respective caecal inocula. In humans, succinate is considered an intermediate metabolite in the global intestinal microbiota fermentation process (Macfarlane and Macfarlane, 2003), since several gut bacteria can convert succinate to propionate or butyrate (Louis and Flint, 2017). In mice, high levels of caecal succinate were reported in response to dietary fiber treatment (Everard et al., 2014) and high succinate levels have been demonstrated to improve glucose metabolism via intestinal gluconeogenesis (De Vadder et al., 2016). When sufficient carbohydrates are present, Bacteroides taxa show reduced need to decarboxylate succinate; thus succinate accumulates instead of propionate (Macy et al., 1978). Furthermore, in Bifidobacteria succinate production is associated with growth (Van Der Meulen et al., 2006), which may explain the high levels detected in our continuous fermentation model due to continuous supply of carbohydrates and therefore growth of these bacteria. The qualitative assessment of the predicted microbial functions by PICRUSt indicated that the gene contents of most pathways were maintained in our model, despite changes in abundances of bacterial populations. These results also suggest that the reactor microbiota as a whole did not change its functional fermentation potential, such as metabolic cross-feeding pathways, from *in vivo* (caecal inoculum) to *in vitro* (reactor).

The *in vitro* murine caecal microbiota was mainly composed of the bacterial phyla Firmicutes and Bacteroidetes, both in the range of *in vivo* caecal microbiota compositions, previously reported (Ley et al., 2006; Clavel et al., 2016; Hugenholtz and de Vos, 2017). However, there was a shift toward higher Bacteroidetes levels compared to caecal inocula; similar shifts were reported for *in vitro* human intestinal microbiota models (Rajilic-Stojanovic et al., 2010; Van den Abbeele et al., 2010; McDonald et al., 2013; Fehlbaum et al., 2015). The high levels of complex carbohydrates in nutritive media used for intestinal fermentation models may favor the growth of Bacteroidetes taxa, since they have a higher glycan-degrading capacity compared to Firmicutes species (Mahowald et al., 2009). The taxa within murine bacterial families that were maintained in the *in vitro* fermentations belong to important functional groups for intestinal fermentation such as primary fibrolytic (*Bacteroidaceae*, *Ruminococcaceae*, *Bifidobacteriaceae*), glycolytic (*Lactobacillaceae, Enterococcaceae, Enterobacteriaceae)* and mucolytic *(Verrucomicrobiaceae, Bacteroidaceae)* bacteria*;* and secondary butyrate- and propionate-producing bacteria (*Lachnospiraceae*, *Ruminococcaceae*, *Erysipelotrichaceae, Rikenellaceae, Verrucomicrobiaceae*) (Chassard and Lacroix, 2013). The prevalent murine intestinal bacterial family *Muribaculaceae* (S24-7) was not maintained in our *in vitro* model. Only recently, Lagkouvardos et al. (2016) succeeded to isolate and cultivate the first strain of this family in a medium containing meat and blood. Interestingly, a recent study reported that this *Muribaculaceae* (S24-7) strain is extremely sensitive to high osmolality (Tropini et al., 2018) and the higher osmolality in our reactors compared to caecum may also explain its low establishment *in vitro*. Further characterization of the physiology and nutritional requirements of strains from the *Muribaculaceae* family and other taxa that were not maintained *in vitro* will be important to further optimize our murine nutritive fermentation medium. Adjustments to nutritive medium, pH and RT helped to control the *in vitro* levels of *Enterobacteriaceae*, which often bloom in fermentation models (Fehlbaum et al., 2015; McDonald et al., 2015) due to their competitive advantage during initial colonization and balancing of the fermentation model.

There was an important overall decrease in bacterial diversity *in vitro* compared to the high caecal inoculum diversity, which were in the range of published Shannon diversity indices ranging from 4.5 to 6 (Holm et al., 2015; Hu et al., 2017). Such effects were also observed in other *in vitro* fermentation models inoculated with high diverse intestinal microbial communities from humans (infants, adult and elderly) (Rajilic-Stojanovic et al., 2010; Van den Abbeele et al., 2010; McDonald et al., 2013; Feria-Gervasio et al., 2014; Poeker et al., 2018) and swine (Tanner et al., 2014). *In vitro* models cannot simulate all the conditions occurring in the host, which are not well-known or cannot be mimicked such as immune response, variation in feed rates and composition, hormonal and digestive secretions (e.g., bile), feedback mechanisms, absorption and peristaltic movements, all of which influence microbial diversity and can only be an approximation of realistic conditions (Lacroix et al., 2015). The well-controlled conditions *in vitro* may result in a loss of redundant species or species thriving on specific host secretions. Murine intestinal bacterial isolation efforts (Lagkouvardos et al., 2016) offer the opportunity to identify species-specific growth requirements and will allow adaptation of fermentation conditions for their improved establishment *in vitro*. Finally, the presence of transient bacteria, i.e., from upper gastrointestinal tract, diet and ingested microbes due to coprophagy, in the caecal inocula may also overestimate the bacterial diversity of the resident caecal community. By mimicking the murine caecal conditions, we obtained a microbial community composed of caecal murine-derived bacteria, with reduced complexity but resulting in a stable and functional caecal fermentation.

Mouse experiments have gained attention as tool to study the gut microbiota in health and disease. However, their poor reproducibility within and between facilities was associated with high variability among the mouse gut microbiota, which makes it hard to draw robust conclusions (Laukens et al., 2015). Factors contributing to the heterogeneity of the mouse gut microbiota within a facility include differences in food intake (Zarrinpar et al., 2014), maternal effects (Hufeldt et al., 2010), hormones (Org et al., 2016), cage (Hildebrand et al., 2013), presence of surrounding animals (Rausch et al., 2016), and other stressors (Bangsgaard Bendtsen et al., 2012) and environmental factors. To control for these factors, our strategy is to uncouple the microbiota from the host and study it under very well-controlled conditions (e.g., fermenter). In contrast to *in vivo* models, *in vitro* models allow study of the dynamics of the complex microbiota following manipulations, particularly to follow production of *in situ* fermentation metabolites, which are partly absorbed *in vivo*. Identified microbes or metabolites that drive microbiota functionality can then be validated further *in vivo*. Moreover, the continuous cultivated *in vitro* murine caecal microbiota can be used as transplantation material to study host–microbiota interactions in murine models of health and disease. This approach is currently performed with pooled murine fecal material, which is less controlled and available, or with bacterial consortia of human- or mouse-derived strains (Clavel et al., 2016). Recently the Oligo-Mouse-Microbiota (Brugiroux et al., 2016), a mixture of up to 15 intestinal murine-derived strains from the mouse intestinal bacterial collection (Lagkouvardos et al., 2016), was established following a bottom-up approach of rational strain selection. Alternatively, with our novel murine caecal fermentation model we can follow a top–down approach of creating functional different murine-derived bacterial communities with higher diversity compared to current consortia.

## Conclusion

We showed that it is feasible to maintain a stable and simplified, but yet representative *in vitro* murine microbiota in a continuous murine caecal fermentation model inoculated with immobilized caecal microbiota. Our simplified, yet representative *in vitro* murine bacterial community showed a similar functionality to inoculum microbiota. We demonstrated that it is feasible to continuously cultivate caecal murine microbiota while maintaining its overall functionality over a long time period. Our model is a first step in the development of a mouse microbiota model system. With the expected increased knowledge of mouse gut isolates, further improvements of our murine *in vitro* model can be carried out by fine-tuning operational or nutritional requirements, and hence to increase preservation of microbial diversity in our model. In addition, our model can be expanded further with second-stage reactors, continuously inoculated with reactor effluent to allow parallel testing of different manipulations on the same microbiota. Hence, our novel *in vitro* model is a promising tool for studying the murine microbe–microbe interactions in response to biotic or abiotic factors that are linked to gut microbial functionality and structure.

## Ethics Statement

Mouse experiments were conducted according to Swiss animal welfare legislation, and the local veterinary office approved all procedures (Veterinäramt des Kantons Zürich; Nr. ZH220/2016).

## Author Contributions

SP, AG, TW, and CL conceived the experiments. SP, AG, and TW conducted the experiments. SP and AG analyzed 16S rRNA sequence, HPLC and qPCR data. SP prepared the figures. SP, AG, MRS, MS, and CL interpreted the results. SP, AG, and CL wrote the manuscript. MRS and MS bred and sacrificed the mice. All authors reviewed the manuscript.

## Conflict of Interest Statement

The authors declare that the research was conducted in the absence of any commercial or financial relationships that could be construed as a potential conflict of interest.
