# Supplementary material for: Stepwise Development of an in vitro Continuous Fermentation Model for the Murine Caecal Microbiota
Source: Front Microbiol. 2019 May 29;10:1166. doi: 10.3389/fmicb.2019.01166 (PMC6548829; doi:10.3389/fmicb.2019.01166)
Supplement: Supplementary file 1 [file Presentation_1.pdf]

SUPPLEMENTARY MATERIAL

Stepwise development of an *in vitro* continuous fermentation model for the murine caecal microbiota

Sophie A. Poeker[1], Christophe Lacroix[1], Tomas de Wouters[1], Marianne R. Spalinger[2], Michael Scharl[2] and Annelies Geirnaert[1*]

[1] Laboratory of Food Biotechnology, Institute of Food, Nutrition and Health, ETH Zurich, Switzerland.

[2] Division of Gastroenterology and Hepatology, University Hospital Zurich, Zurich, Switzerland.

* Corresponding author: Annelies Geirnaert, Laboratory of Food Biotechnology, Institute of Food, Nutrition and Health, ETH Zurich, Schmelzbergstrasse 7, 8092 Zürich, Switzerland; Phone: +41 44 632 14 03; mail: annelies.geirnaert@hest.ethz.ch

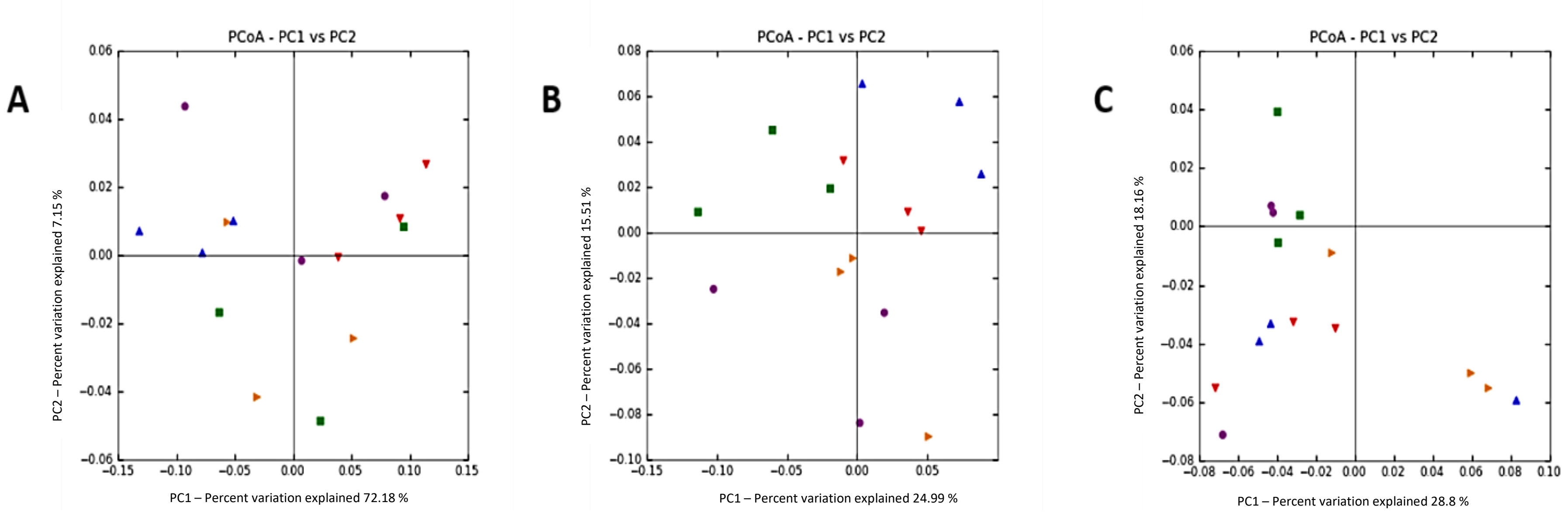

Supplementary Figure S1*:* Individual variations among the caecal murine microbial communities. PCoA showing caecal microbiota from WT C57BL/6 mice housed in different cages based on weighted (A), unweighted (B) and generalized (C) UniFrac distance matrix. N=3 per cage; cage 1: red; cage 2: blue; cage 3: orange; cage 4: green; cage 5: purple

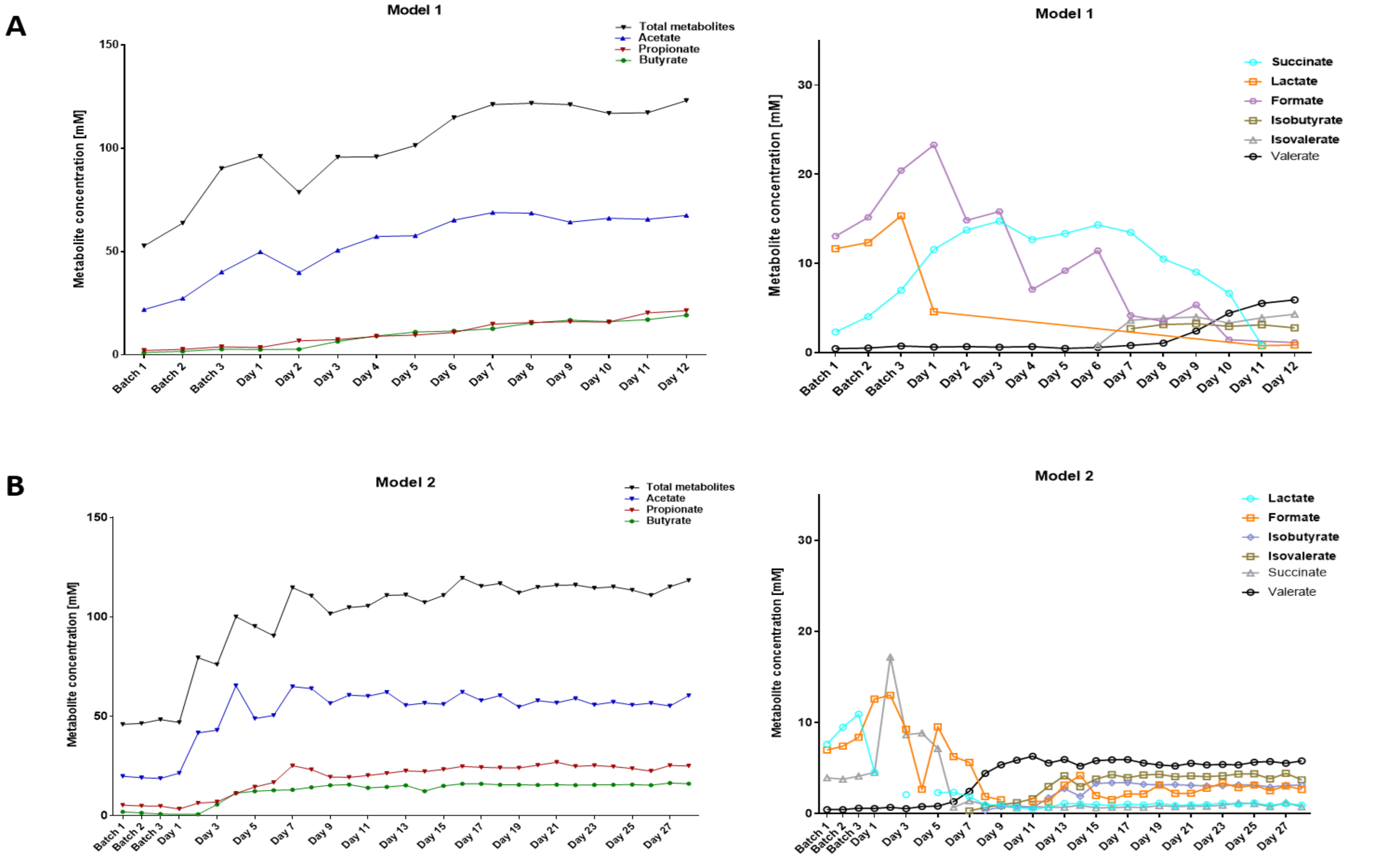

Supplementary Figure S2: Daily fermentation metabolite concentrations in reactor effluents of models 1 and 2 (A & B) measured by HPLC. Left the end metabolites (acetate, propionate and butyrate), and right the intermediate metabolites (formate, lactate and succinate) and branched-chain fatty acids (isobutyrate, isovalerate) and valerate. Colonization: three consecutive fed-batch fermentations for bead colonization.

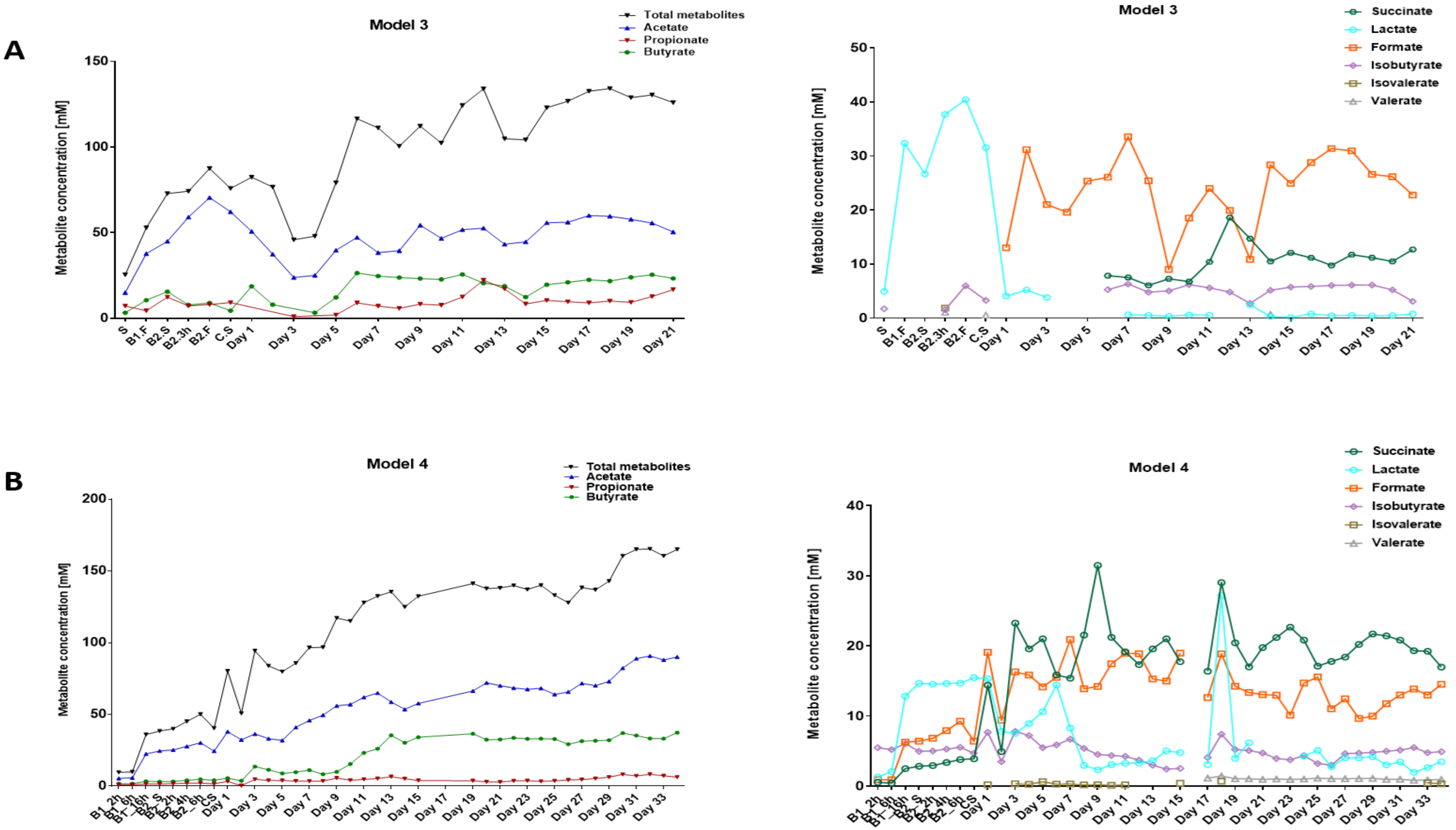

Supplementary Figure 3: Daily fermentation metabolite concentrations in reactor effluents of models 3 and 4 (A & B) measured by HPLC. Left the end metabolites (acetate, propionate and butyrate), and right the intermediate metabolites (formate, lactate and succinate) and branched-chain fatty acids (isobutyrate, isovalerate) and valerate. Colonization: three consecutive fed-batch fermentations for bead colonization.

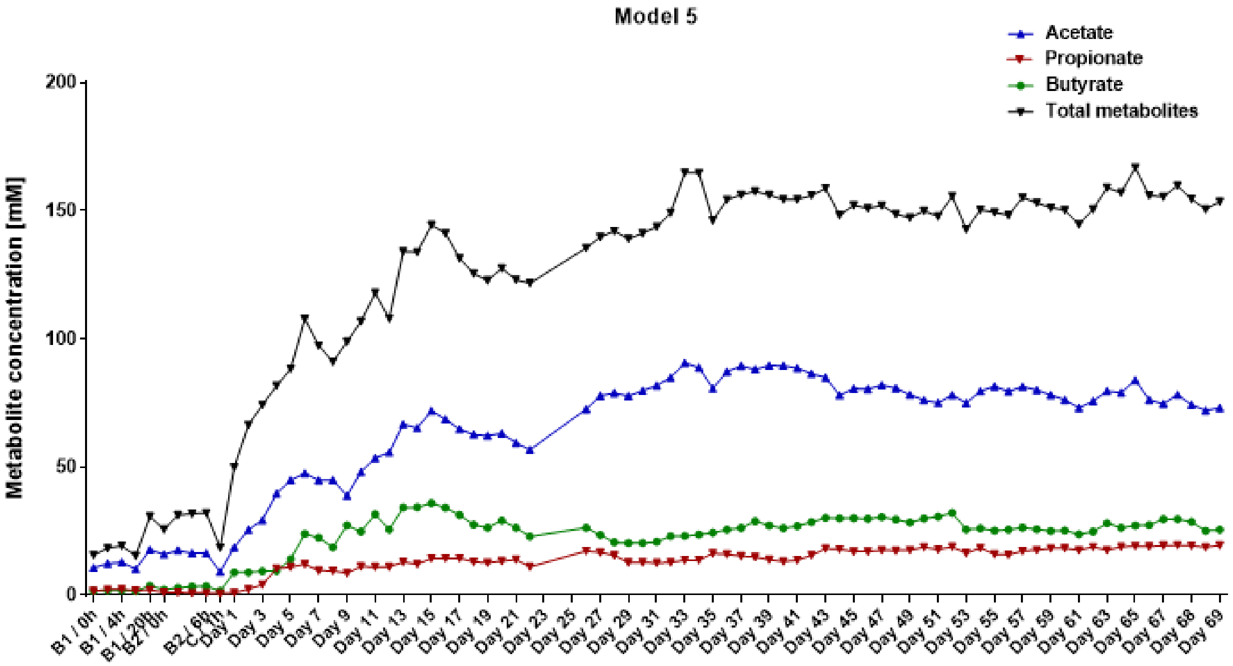

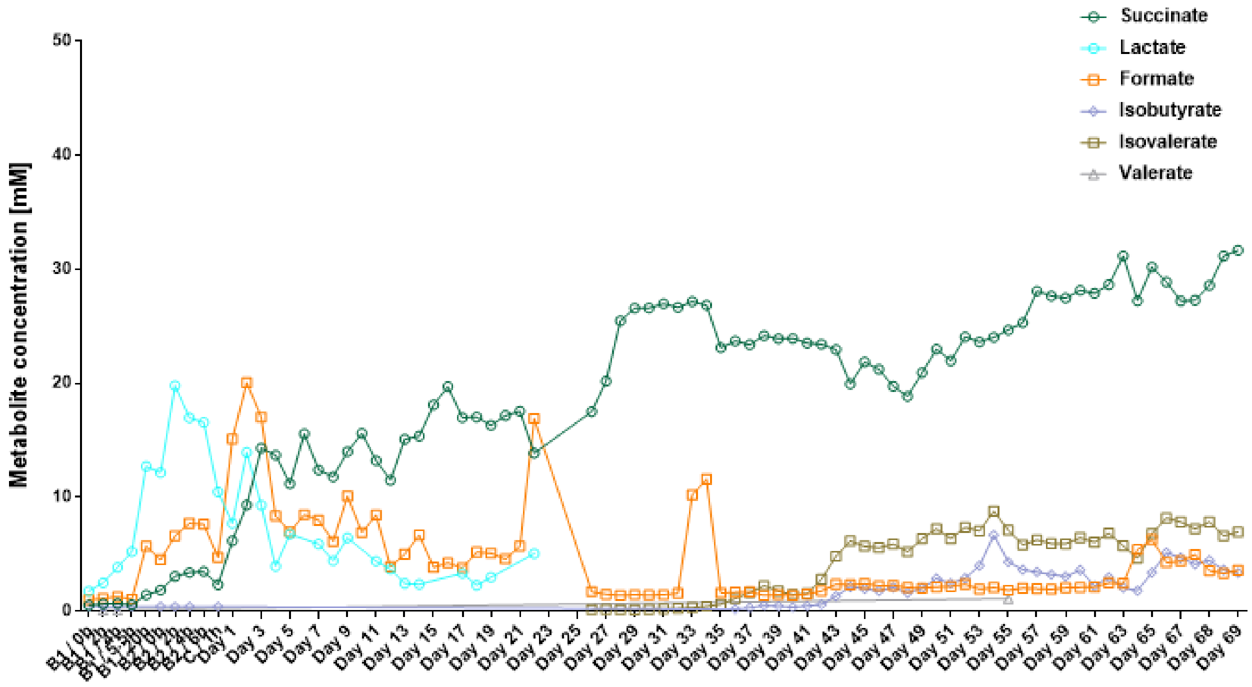

Supplementary Figure S4: Daily fermentation metabolite concentrations in reactor effluents of models 5 measured by HPLC. On top the end metabolites (acetate, propionate and butyrate), and below the intermediate metabolites (formate, lactate and succinate) and branched-chain fatty acids (isobutyrate, isovalerate) and valerate. Colonization: three consecutive fed-batch fermentations for bead colonization.

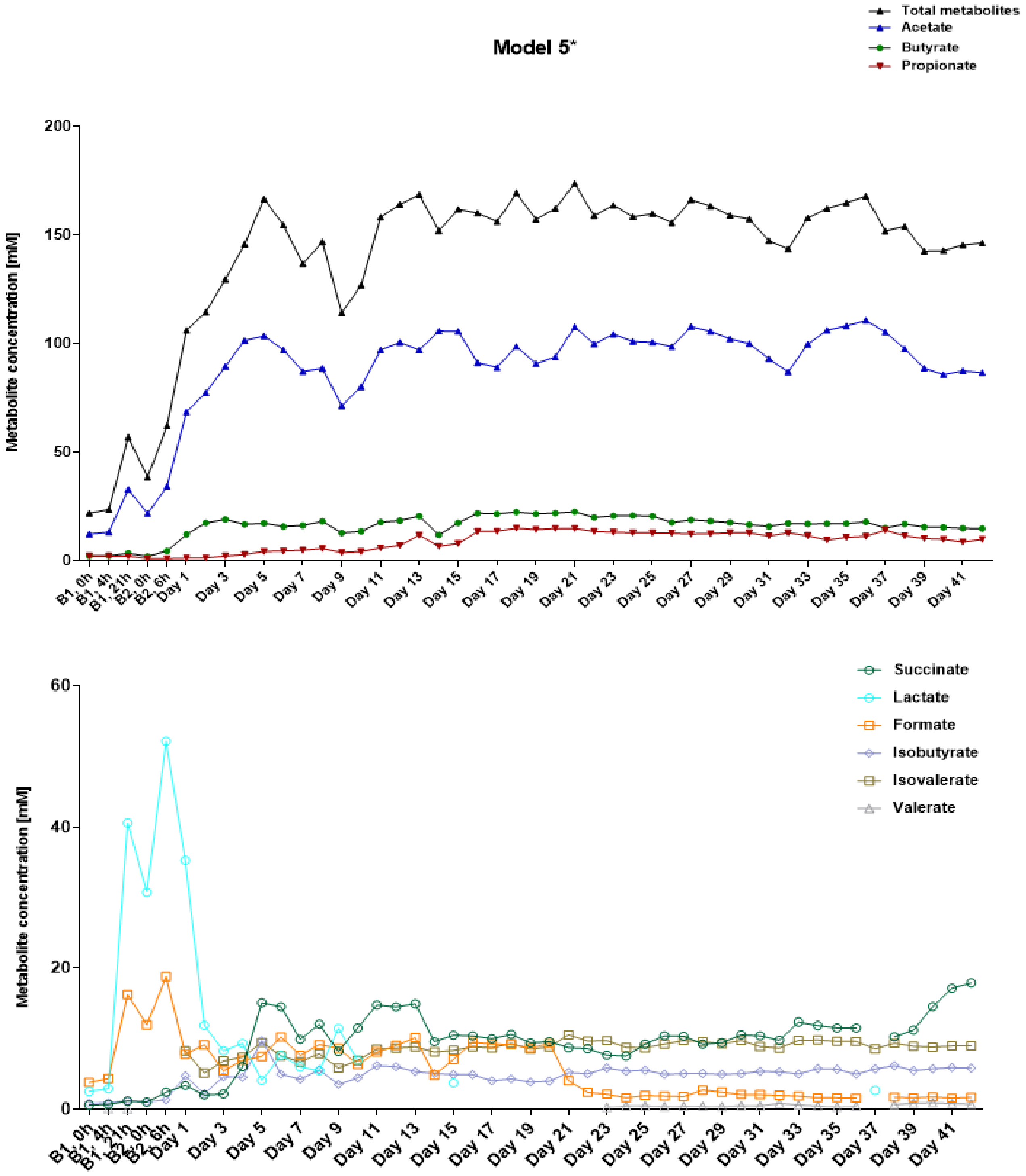

Supplementary Figure S5: Daily fermentation metabolite concentrations in reactor effluents of models 5* measured by HPLC. On top the end metabolites (acetate, propionate and butyrate), and below the intermediate metabolites (formate, lactate and succinate) and branched-chain fatty acids (isobutyrate, isovalerate) and valerate. Colonization: three consecutive fed-batch fermentations for bead colonization.

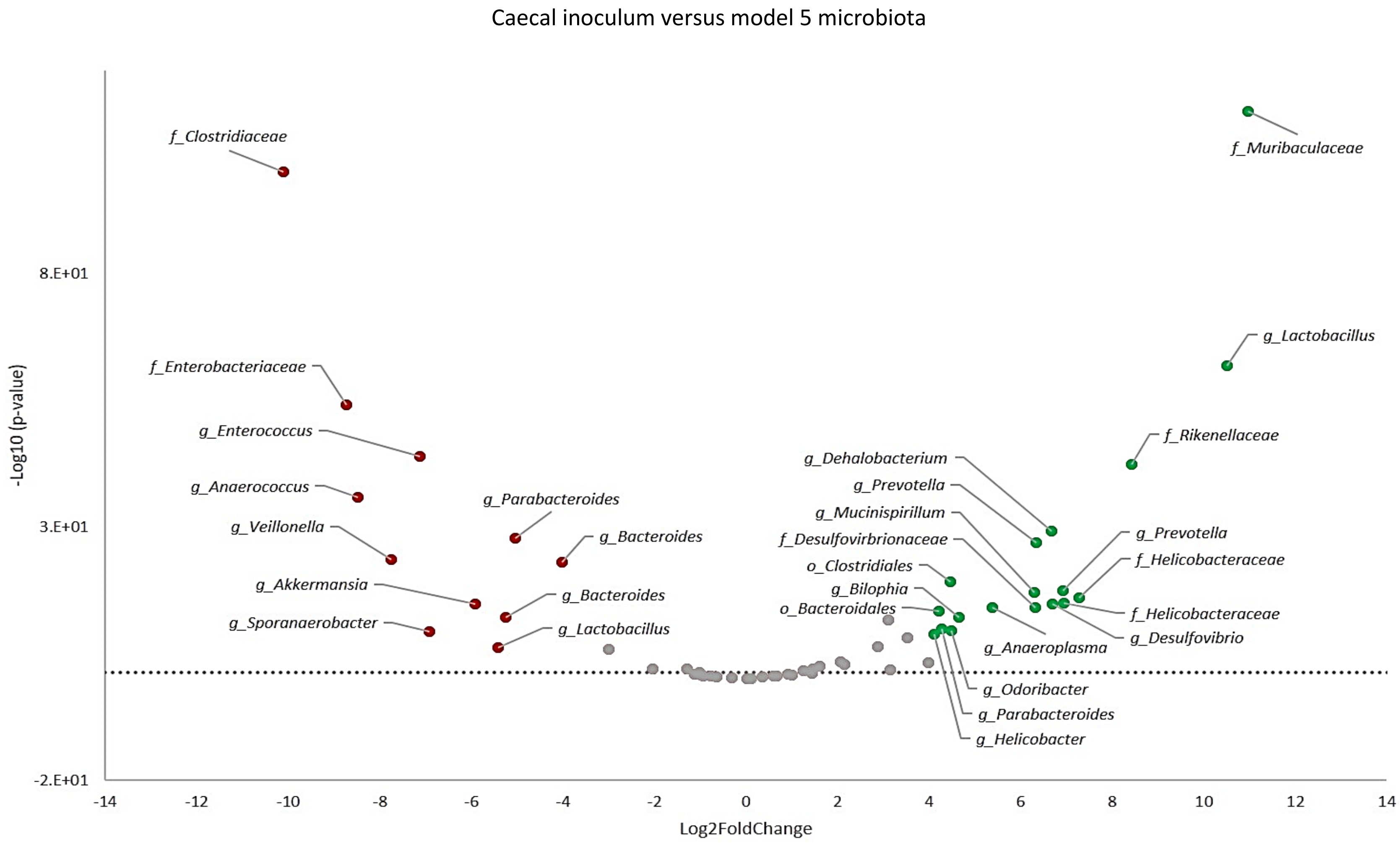

Supplementary Figure S6: Microbiota plots illustrating OTUs that were significantly enriched (green) and reduced (red) in reactor effluent of model 5 compared to the caecal inoculum as determined by differential abundance analysis. Each point represents an individual OTU, and the Y-axis indicates the Log2 fold change of relative abundance. The dashed line represents the statistically p-value of 0.05.

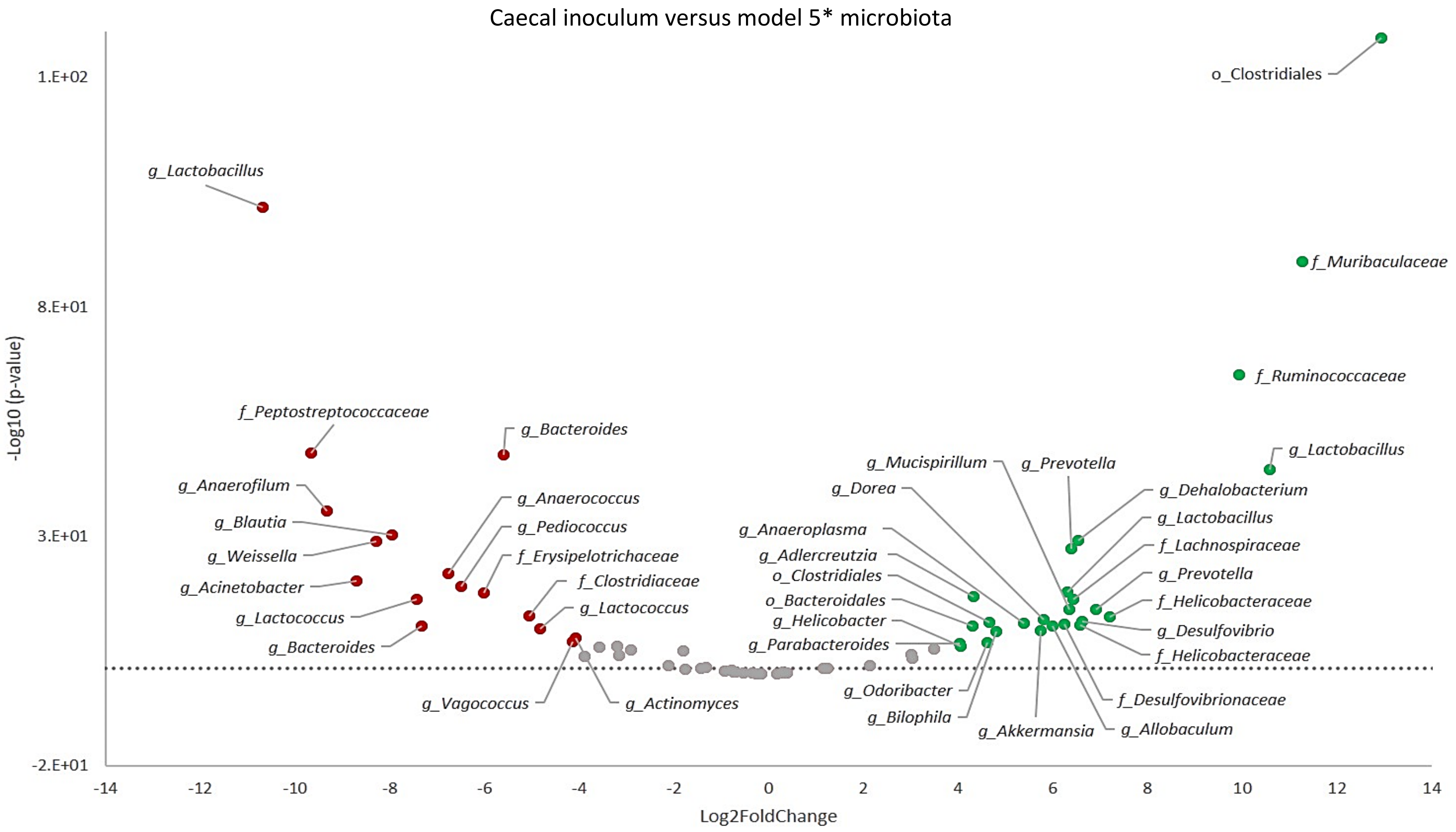

Supplementary Figure S7: Microbiota plots illustrating OTUs that were significantly enriched (green) and reduced (red) in reactor effluent of model 5* compared to the caecal inoculum as determined by differential abundance analysis. Each point represents an individual OTU, and the Y-axis indicates the Log2 fold change of relative abundance. The dashed line represents the statistically p-value of 0.05.

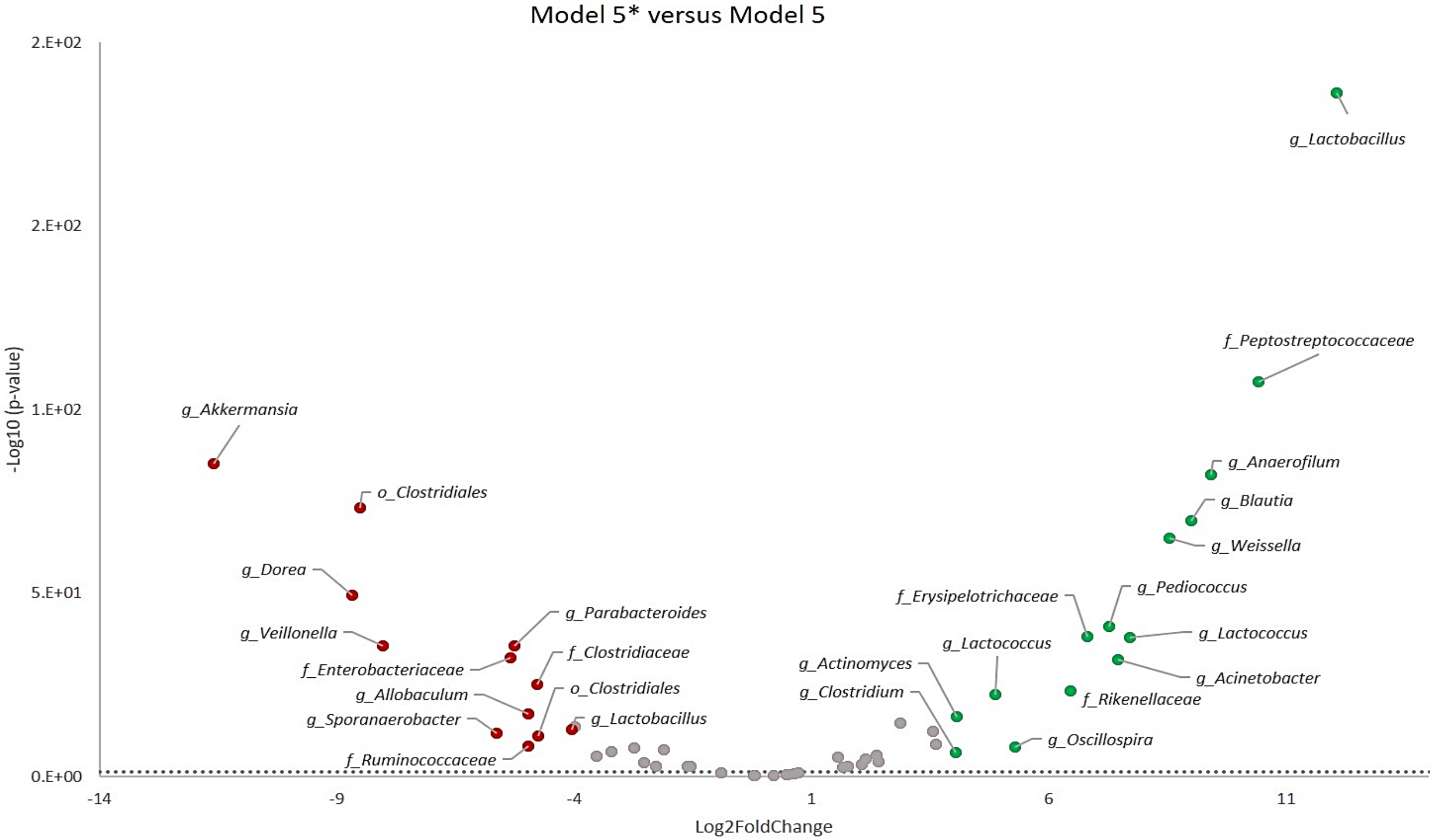

Supplementary Figure S8: Microbiota plots illustrating OTUs that were significantly enriched (green) and reduced (red) in reactor effluent of model 5 versus reactor effluent of model 5* as determined by differential abundance analysis. Each point represents an individual OTU, and the Y-axis indicates the Log2 fold change of relative abundance. The dashed line represents the statistically p-value of 0.05.

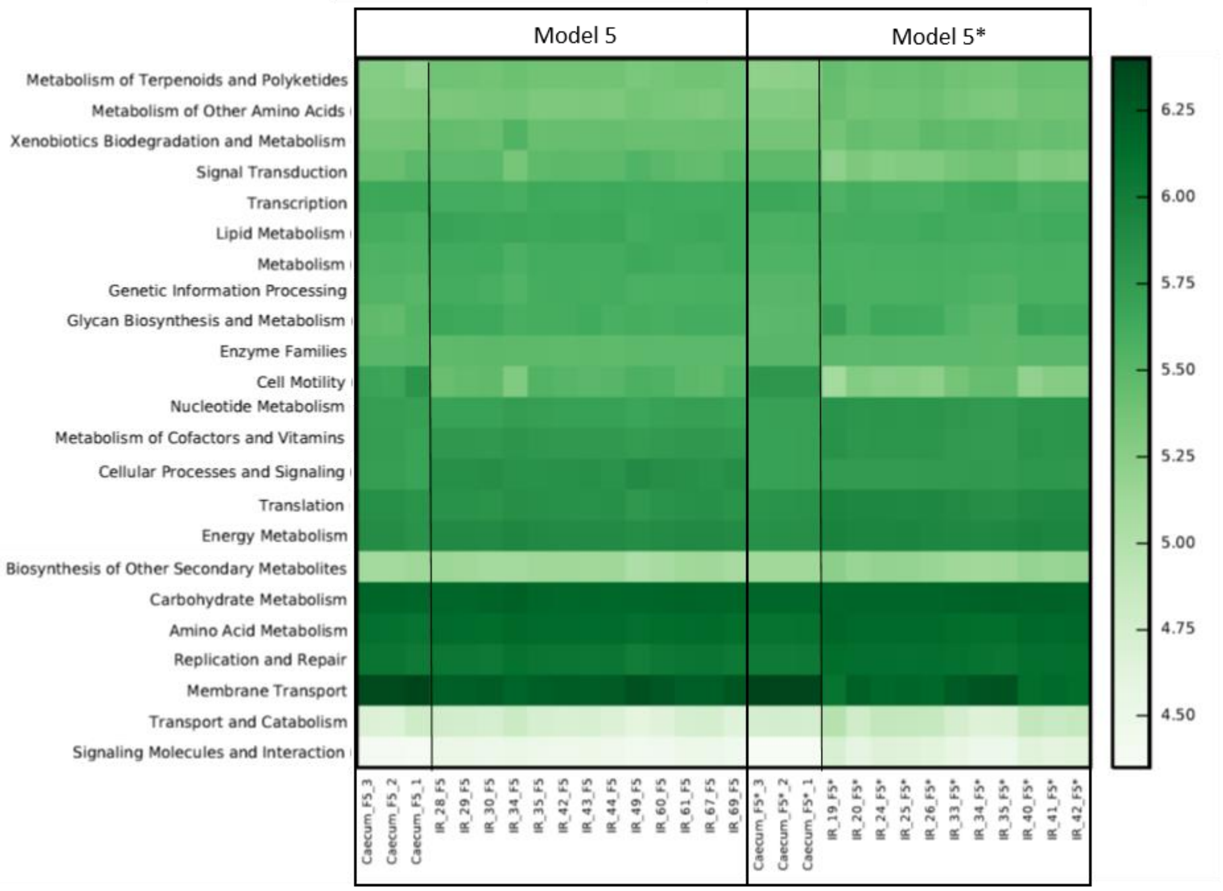

Supplementary Figure S9: Predictive functional profiling of microbial communities of caecal inocula and model 5 and model 5* (during stabilization) by PICRUSt. Heatmap depicting the log transformed gene abundance of microbiota-associated predicted KEGG pathways. Numbers in scale represent log range of gene abundances for this dataset.

Supplementary Table S1: Composition of murine nutritive growth medium (g/L) (A) and of three nutritive mouse chows (B).

A

	Medium 1	Medium 2
Constituent	g/l	g/l
Pectine (citrus)	2	2
Xylane (oat spelts)	4.8	4.8
Arabinogalactan (larch wood)	4.8	4.8
Soluble starch (corn)	6.8	2
Mucine	4	4
Casein acid hydrolysate	3.6	3.6
Peptone water	6	6
Bacto™ Tryptone	6	6
Yeast extract	5.4	5.4
L-Cystein HCl	0.8	0.8
Bile salts (adult formulation: 0.4)	0.4	0.4
KH_2PO_4	0.5	0.5
$NaHCO_3$	1.5	1.5
NaCl	4.5	4.5
KCl	4.5	4.5
$MgSO_4$ anhydrous (M: 120.37)	0.61	0.61
$CaCl_2 \cdot 2H_2O$ (M: 147.02)	0.1	0.1
$MnCl_2 \cdot 4H_2O$ (M: 197.91)	0.2	0.2
$FeSO_4 \cdot 7H_2O$ (M: 278.02)	0.005	0.005
Hemin solution	0.05	0.05
Tween 80	1 ml	1 ml
Vitamin solution	1 ml	1 ml

B

KLIBA NAFAG 3430 Mouse and Rat		
Carbohydrates	58.7%	approx 68 % fibers
Proteins	18.5%	
Fat/ash	10.8%	

Supplementary Table S2: Primers used for the enumeration of specific bacterial populations in caecal and effluent samples by qPCR analysis

Name	Sequence 5'-3'	Target gene	Reference
Eub 338F	ACT CCT ACG GGA GGC AGC AG	Total bacteria	Guo et al., 2008
Eub 518R	ATT ACC GCG GCT GCT GG		
Firm 934F	GGA GYA TGT GGT TTA ATT CGA AGC A	Firmicutes	Guo et al., 2008
Firm 1060R	AGC TGA CGA CAA CCA TGC AC		
Clep 866mF	TTA ACA CAA TAA GTW ATC CAC CTG G	*Clostridium* Cluster IV	Ramirez-Farias et al., 2009
Clep 1240mR	ACC TTC CTC CGT TTT GTC AAC		
Clep 14aF	AAA TGA CGG TAC CTG ACT AA	*Clostridium* Cluster XIVa	Matsuki et al., 2002
Clep 14aR	CTT TGA GTT TCA TTC TTG CGA A		
Bac 303F	GAA GGT CCC CCA CAT TG	Bacteroidetes	Bartosch et al., 2004
Bact Pre-rev	CTT TGA GTT TCA CCG TTG CCG G		
Bac 303F	GAA GGT CCC CCA CAT TG	*Bacteroides* spp.	Ramirez-Farias et al., 2009
Bfr-Femrev	CGC KAC TTG GCT GGT TCA G		
Eco 1457F	CAT TGA CGT TAC CCG CAG AAG AAG C	*Enterobacteriaceae*	Bartosch et al., 2004
Eco 1652R	CTC TAC GAG ACT CAA GCT TGC		
F_Lacto 05	AGC AGT AGG GAA TCT TCC A	*Lactobacillus* | *Leuconostoc* | *Pediococcus* spp.	Furet et al., 2009
R_Lacto 04	CGC CAC TGG TGT TCY TCC ATA TA		
AM1	CAG CAC GTG AAG GTG GGG AC	*Akkermanisa* spp.	Collado et al., 2007
AM2	CCT TGC GGT TGG CTT CAG AT		

Supplementary Table S3: Mean metabolite concentrations with standard error of effluent samples in reactor effluents during stabilization phase and caecal contents (n=15).

	Concentrations (mM)									Ratios (%)			Days
	Formate	Acetate	Propionate	Butyrate	Valerate	Lactate	Succinate	BCFAs	Total metabolites	Acetate	Propionate	Butyrate	
**Model 1**													
Caecal content[1]	3.5	40.2	3.9	3.6	ND	12.0	4.5	ND	55.7	83.3	8.3	8.3	
IR[2]	5.4 ± 3.6	64.6 ± 4.3	14.9 ± 4.4	14.4 ± 3.4	2.4 ± 2.3	0.8 ± 0.1	10.1 ± 4.6	4.6 ± 3.3	114.8 ± 9.7	69.1	16.0	14.9	d4-12
**Model 2**													
Caecal content[1]	ND	30.2	5.6	4.6	ND	22.2	8.4	ND	48.8	73.2	14.6	12.2	
IR[2]	2.6 ± 0.7	57.6 ± 2.4	24.1 ± 1.4	15.3 ± 0.9	5.8 ± 0.2	1.0 ± 0.1	0.8 ± 0.2	7.0 ± 0.9	114.0 ± 3.2	60.6	24.2	15.2	d12-28
**Model 3**													
Caecal content[1]	ND	59.8	ND	46.7	ND	ND	28.0	0.8	88.6	56.1	ND	43.9	
IR[2]	17.8 ± 9.0	56.5 ± 3.2	11.2 ± 2.8	22.5 ± 1.9	ND	0.5 ± 0.2	11.3 ± 1.0	5.5 ± 1.1	134.8 ± 4.4	62.6	12.1	25.3	d15-21
**Model 4**													
Caecal content[1]	ND	73.2	15.4	82.5	0.7	1.5	9.1	152.6	335.0	42.7	8.8	48.5	
IR[2]	12.7 ± 1.8	74.8 ± 9.6	5.1 ± 1.9	33.4 ± 2.2	1.0 ± 0.0	3.8 ± 1.0	19.7 ± 1.7	2.5 ± 0.4	154.3 ± 13.4	66.4	4.4	29.2	d19-34
**Model 5**													
Caecal inoculum[1]	ND	88.0	12.8	39.9	0.8	5.2	5.3	ND	152.0	62.4	9.2	28.4	
IR[2]	2.7 ± 2.1	80.4 ± 5.1	16.8 ± 2.1	26.6 ± 3.0	ND	ND	25.1 ± 3.4	3.5 ± 2.2	158.8 ± 8.9	64.5	13.7	21.8	d26-69
**Model 5***													
Caecal inoculum[1]	1.2	33.2	5.9	11.8	0.5	3.5	6.5	ND	59.1	64.7	11.8	23.5	
IR[2]	3.8 ± 3.1	98.5 ± 7.5	12.0 ± 1.4	18.2 ± 2.7	ND	1.3 ± 0.5	10.5 ± 3.1	14.4 ± 1.1	160.9 ± 7.4	76.7	9.3	14.0	d13-42

[1] extracted from 100 mg of caecal content

[2] Mean and standard error reported over whole stabilized fermenation time

Supplementary Table S4: qPCR quantification of bacterial populations in caecal inocula and reactor effluent samples of different models at the end of the stabilization phase.

	Total 16S rRNA gene	Firmicutes	*Ruminococcaceae*	*Lachnospiraceae*	Bacteroidetes	Bacteroides spp.	*Enterobacteriaceae*	*Lactobacillus spp.*	*Akkermansia spp.*	Days
**Model 1**										
Caecal inoculum[1]	11.9	11.0	9.6	9.0	10.9	10.4	7.7	9.7	4.9	
IR[3]	10.7	9.4	8.4	8.4	10.4	10.2	9.2	7.3	4.6	8
**Model 2**										
Caecal inoculum[1]	11.8	10.8	9.2	8.9	11.0	10.3	8.1	9.5	4.8	
IR[4]	10.7 ± 0.2	9.4 ± 0.2	8.6 ± 0.3	8.4 ± 0.3	10.1 ± 0.4	9.0 ± 0.3	8.8 ± 0.5	6.4 ± 0.1	4.7 ± 0.1	26-28
**Model 3**										
Caecal inoculum[1]	11.2	11.0	9.0	10.6	10.3	9.9	6.2	8.7	5.0	
IR[4]	11.2 ± 0.3	10.8 ± 0.3	BDL	9.2 ± 0.3	10.8 ± 0.2	9.8 ± 0.1	10.1 ± 0.2	6.9 ± 0.4	4.6 ± 0.1	17-19
**Model 4**										
Caecal inoculum[1]	11.6	11.4	7.8	10.5	10.5	9.5	6.0	9.3	8.1	
IR[4]	11 ± 0.1	10.3 ± 0.1	7.7 ± 0.1	9.4 ± 0.3	10.0 ± 0.3	9.7 ± 0.3	9.8 ± 0.7	BDL	9.5 ± 0.5	27-29
**Model 5**										
Caecal inoculum[2]	12.1 ± 0.0	11.1 ± 0.0	8.3 ± 0.0	10.1 ± 0.0	10.8 ± 0.2	7.5 ± 0.3	5.7 ± 0.0	8.4 ± 0.1	8.6 ± 0.1	
IR[4]	11.5 ± 0.5	10.3 ± 0.1	7.1 ± 0.3	8.2 ± 0.3	10.6 ± 0.2	10.0 ± 0.1	7.7 ± 0.1	6.6 ± 0.1	8.6 ± 0.4	29, 30 & 36
**Model 5***										
Caecal inoculum[2]	11.7 ± 0.2	11.1 ± 0.2	9.1 ± 0.1	10.4 ± 0.0	10.4 ± 0.2	9.3 ± 0.1	6.3 ± 0.2	8.2 ± 0.2	6.7 ± 0.1	
IR[4]	11.4 ± 0.1	10.4 ± 0.1	7.5 ± 0.1	8.8 ± 0.2	9.7 ± 0.5	9.2 ± 0.5	6.6 ± 0.3	8.5 ± 0.6	5.7 ± 0.1	18-20

[1] Data are mean log_{10} copies 16S rRNA gene g^{-1} of caecal inoculum used for fermentation

[2] Data are mean log_{10} copies 16S rRNA gene g^{-1} ± SD of caecal inoculum (extracted twice) used for fermentation

[3] Data are mean log10 copies 16S rRNA gene mL^{-1} of one day at the end of the stabilization period

[4] Data are mean log10 copies 16S rRNA gene mL^{-1} ± SD of one day at the end of the stabilization period extracted in duplicate; samples were analyzed in duplicate

BDL of 4.0 log_{10} copies per mL

Supplementary Table S5 – Summary microbial phyla and most abundant (> 1 %) bacterial families obtained by V4 region 16S amplicon sequencing within PolyFermS reactors of model 5 and 5*. Values < 1 % are summarized in the group «Others».

Taxon	Caecal inoculum 5	IR Model 5	Caecal inoculum 5*	IR Model 5*
**Actinobacteria**	**0.7% ± 0.5%**	**0.1% ± 0.1%**	**0.3% ± 0.1%**	**0.4% ± 0.9%**
Actinomycetaceae	0.0% ± 0.0%	0.0% ± 0.0%	0.0% ± 0.0%	0.3% ± 0.7%
Microbacteriaceae	0.0% ± 0.0%	0.0% ± 0.0%	0.0% ± 0.0%	0.0% ± 0.0%
Bifidobacteriaceae	0.3% ± 0.6%	0.0% ± 0.0%	0.1% ± 0.0%	0.1% ± 0.1%
Coriobacteriaceae	0.4% ± 0.1%	0.1% ± 0.1%	0.3% ± 0.0%	0.1% ± 0.1%
**Bacteroidetes**	**11.1% ± 3.1%**	**29.1% ± 6.4%**	**9.1% ± 1.7%**	**64.7% ± 14.2%**
UC Bacteroidales	0.1% ± 0.1%	0.0% ± 0.0%	0.0% ± 0.0%	0.0% ± 0.0%
Bacteroidaceae	0.9% ± 0.5%	24.4% ± 7.3%	2.6% ± 0.5%	64.3% ± 14.3%
Porphyromonadaceae	0.0% ± 0.0%	4.7% ± 2.1%	0.4% ± 0.2%	0.2% ± 0.1%
Prevotellaceae	0.2% ± 0.2%	0.0% ± 0.0%	0.2% ± 0.1%	0.0% ± 0.0%
Rikenellaceae	1.4% ± 0.4%	0.0% ± 0.0%	0.6% ± 0.7%	0.2% ± 0.2%
Muribaculaceae	8.2% ± 2.1%	0.0% ± 0.0%	4.7% ± 1.0%	0.0% ± 0.0%
Odoribacteraceae	0.1% ± 0.1%	0.0% ± 0.0%	0.0% ± 0.0%	0.0% ± 0.0%
Paraprevotellaceae	0.1% ± 0.1%	0.0% ± 0.0%	0.6% ± 0.4%	0.0% ± 0.0%
Deferribacteraceae	0.1% ± 0.1%	0.0% ± 0.0%	0.3% ± 0.3%	0.0% ± 0.0%
**Firmicutes**	**86.9% ± 3.3%**	**40.0% ± 7.5%**	**86.7% ± 4.0%**	**31.8% ± 12.5%**
Planococcaceae	0.0% ± 0.0%	0.0% ± 0.0%	0.0% ± 0.0%	0.1% ± 0.2%
Enterococcaceae	0.0% ± 0.0%	0.8% ± 0.3%	0.0% ± 0.0%	0.1% ± 0.1%
Lactobacillaceae	5.6% ± 5.9%	0.2% ± 0.2%	2.0% ± 0.8%	11.5% ± 4.8%
Leuconostocaceae	0.0% ± 0.0%	0.0% ± 0.0%	0.0% ± 0.0%	0.8% ± 0.5%
Streptococcaceae	0.0% ± 0.0%	0.0% ± 0.0%	0.0% ± 0.0%	0.4% ± 0.5%
UC Clostridiales	57.8% ± 10.9%	2.0% ± 1.3%	62.1% ± 4.6%	0.0% ± 0.0%
Clostridiaceae	0.1% ± 0.0%	28.7% ± 8.0%	0.0% ± 0.0%	0.9% ± 0.8%
Dehalobacteriaceae	0.4% ± 0.3%	0.0% ± 0.0%	0.2% ± 0.1%	0.0% ± 0.0%
Eubacteriaceae	0.0% ± 0.0%	0.0% ± 0.0%	0.0% ± 0.0%	0.0% ± 0.0%
Lachnospiraceae	19.6% ± 12.4%	3.6% ± 1.3%	13.7% ± 1.2%	8.5% ± 5.3%
Peptostreptococcaceae	0.0% ± 0.0%	0.0% ± 0.0%	0.0% ± 0.0%	3.3% ± 2.2%
Ruminococcaceae	3.3% ± 0.9%	1.4% ± 0.7%	6.4% ± 1.5%	5.5% ± 3.1%
Veillonellaceae	0.0% ± 0.0%	0.8% ± 0.5%	0.0% ± 0.0%	0.0% ± 0.0%
Mogibacteriaceae	0.1% ± 0.0%	0.0% ± 0.0%	0.0% ± 0.0%	0.0% ± 0.0%
Tissierellaceae	0.0% ± 0.0%	2.0% ± 1.1%	0.0% ± 0.0%	0.4% ± 0.4%
Erysipelotrichaceae	0.1% ± 0.1%	0.6% ± 0.4%	2.2% ± 0.6%	0.3% ± 0.2%
**Proteobacteria**	**0.2% ± 0.0%**	**10.5% ± 6.7%**	**3.5% ± 1.9%**	**3.1% ± 2.0%**
Alcaligenaceae	0.1% ± 0.0%	0.5% ± 0.6%	0.5% ± 0.3%	1.8% ± 1.3%
Desulfovibrionaceae	0.1% ± 0.0%	0.0% ± 0.0%	1.1% ± 0.3%	0.0% ± 0.0%
Helicobacteraceae	0.0% ± 0.0%	0.0% ± 0.0%	1.8% ± 1.4%	0.0% ± 0.0%
Enterobacteriaceae	0.0% ± 0.0%	10.0% ± 6.6%	0.0% ± 0.0%	0.3% ± 0.3%
Moraxellaceae	0.0% ± 0.0%	0.0% ± 0.0%	0.0% ± 0.0%	1.1% ± 1.5%
**Verrucomicrobia**	**0.8% ± 0.7%**	**20.3% ± 7.8%**	**0.0% ± 0.0%**	**0.0% ± 0.0%**
Verrucomicrobiaceae	0.8% ± 0.7%	20.3% ± 7.8%	0.0% ± 0.0%	0.0% ± 0.0%

References

Bartosch S, Fite A, Macfarlane GT, McMurdo ME. (2004). Characterization of bacterial communities in feces from healthy elderly volunteers and hospitalized elderly patients by using real-time PCR and effects of antibiotic treatment on the fecal microbiota. *Appl Environ Microbiol* **70:** 3575-3581.

Collado MC, Calabuig M, Sanz Y. (2007). Differences between the fecal microbiota of coeliac infants and healthy controls. *Curr Issues Intest Microbiol* **8:** 9-14.

Furet JP, Firmesse O, Gourmelon M, Bridonneau C, Tap J, Mondot S *et al.* (2009). Comparative assessment of human and farm animal faecal microbiota using real-time quantitative PCR. *FEMS Microbiol Ecol* **68:** 351-362.

Guo X, Xia X, Tang R, Zhou J, Zhao H, Wang K. (2008). Development of a real-time PCR method for Firmicutes and Bacteroidetes in faeces and its application to quantify intestinal population of obese and lean pigs. Lett Appl Microbiol 47: 367-373.

Matsuki, T., et al., Development of 16S rRNA-gene-targeted group-specific primers for the detection and identification of predominant bacteria in human feces. Applied and environmental microbiology, 2002. 68(11): p. 5445-5451.

Ramirez-Farias C, Slezak K, Fuller Z, Duncan A, Holtrop G, Louis P. (2009). Effect of inulin on the human gut microbiota: stimulation of Bifidobacterium adolescentis and Faecalibacterium prausnitzii. *Brit J Nutr* **101:** 541-550.
